# Exome Sequencing for Prenatal Detection of Genetic Abnormalities in Fetal Ultrasound Anomalies: An Economic Evaluation

**DOI:** 10.1159/000504976

**Published:** 2020-01-21

**Authors:** Shahela S. Kodabuckus, Elizabeth Quinlan-Jones, Dominic J. McMullan, Eamonn R. Maher, Matthew E. Hurles, Pelham M. Barton, Mark D. Kilby

**Affiliations:** ^a^ Health Economics Unit, Institute of Applied Health Research, College of Medical and Dental Sciences, University of Birmingham, Birmingham, United Kingdom; ^b^ Birmingham Women's and Children's Foundation Trust, Birmingham, United Kingdom; ^c^ West Midlands Regional Genetics Laboratory, Birmingham Women's and Children's Foundation Trust, Birmingham, United Kingdom; ^d^ Department of Medical Genetics, University of Cambridge and NIHR Cambridge Biomedical Research Centre, Cambridge, United Kingdom; ^e^ Wellcome Trust Sanger Institute, Wellcome Trust Genome Campus, Hinxton, United Kingdom; ^f^ Institute of Metabolism and Systems Research, College of Medical and Dental Sciences, University of Birmingham, Birmingham, United Kingdom; ^g^ Centre for Women's and Children's Health, Birmingham Health Partners, Birmingham, United Kingdom

**Keywords:** Exome sequencing, Chromosomal microarray, Fetal anomaly, Economic evaluation

## Abstract

**Introduction:**

In light of the prospective Prenatal Assessment of Genomes and Exomes (PAGE) study, this paper aimed to determine the additional costs of using exome sequencing (ES) alongside or in place of chromosomal microarray (CMA) in a fetus with an identified congenital anomaly.

**Methods:**

A decision tree was populated using data from a prospective cohort of women undergoing invasive diagnostic testing. Four testing strategies were evaluated: CMA, ES, CMA followed by ES (“stepwise”); CMA and ES combined.

**Results:**

When ES is priced at GBP 2,100 (EUR 2,407/USD 2,694), performing ES alone prenatally would cost a further GBP 31,410 (EUR 36,001/USD 40,289) per additional genetic diagnosis, whereas the stepwise would cost a further GBP 24,657 (EUR 28,261/USD 31,627) per additional genetic diagnosis. When ES is priced at GBP 966 (EUR 1,107/USD 1,239), performing ES alone prenatally would cost a further GBP 11,532 (EUR 13,217/USD 14,792) per additional genetic diagnosis, whereas the stepwise would cost a further additional GBP 11,639 (EUR 13,340/USD 14,929) per additional genetic diagnosis. The sub-group analysis suggests that performing stepwise on cases indicative of multiple anomalies at ultrasound scan (USS) compared to cases indicative of a single anomaly, is more cost-effective compared to using ES alone.

**Discussion/Conclusion:**

Performing ES alongside CMA is more cost-effective than ES alone, which can potentially lead to improvements in pregnancy management. The direct effects of test results on pregnancy outcomes were not examined; therefore, further research is recommended to examine changes on the projected incremental cost-effectiveness ratios.

## Introduction

Structural anomalies can be identified prenatally in 3% of pregnancies using ultrasound scan (USS) [1]. They can occur de novo or secondary to chromosomal abnormalities (a change in the structure or number of chromosomes in the DNA) or single gene abnormalities (a mutation in a single gene) that may contribute to long-term disabil­ities and are associated with a risk of perinatal mortality and morbidity. Data collected by the National Congeni­tal Anomaly and Rare Disease Registration Service (NCARDRS) and summarised by the Department of Health (2018) found that in 2016, 205 per 10,000 births were associated with one or more congenital anomaly [2]. It is therefore highly relevant to identify cases prenatally to better understand the genetic aetiology of congenital anomalies and to improve individualised pregnancy management.

Prenatal fetal chromosomal analysis is used to detect the most common causes of congenital anomaly and provides a diagnosis in up to 40% of cases [3]. Such analysis can be performed on samples obtained invasively (i.e., chorionic villus sampling or amniocentesis). Once a fetal DNA sample has been collected, some chromosomal abnormalities may be examined by quantitative fluorescent polymerase chain reaction (QF-PCR) and/or chromosomal microarray (CMA) [4, 5]. Many healthcare systems, such as many regions of the UK, use CMA as a supplement to QF-PCR following a normal genetic diagnosis (where a congenital anomaly has not been identified), whilst some countries (i.e., Belgium) use CMA as a first tier for prenatal indications [6, 7]. Recent studies have found CMA alone to be more effective compared to QF-PCR alone and other conventional testing techniques (e.g., karyotype), with CMA detecting approximately 3–7% more anomalies [8, 9].

Exome sequencing (ES) is a next-generation technology assay that is able to detect single nucleotide variants that are associated with monogenic disorders (disorders caused by a mutation in a single gene) [10, 11]. In our recently published prospective multicentre study from the UK, using prenatal ES in fetuses with structural anomalies on ultrasound found that anomalies were identified in 52/610 cases (8.5; 95% CI 6.4–11.0%) and a further 24 cases (3.9%) were identified as variant of uncertain significance (VUS) with potential clinical utility [12]. This potentially allows better prognostic prediction; including the prediction of recurrence risks.

ES is an additional health economic expense as for optimal diagnostic yield trio samples (fetus, mother, and father) are required. Nonetheless, given the increased detection rate for monogenic disorders, the technique is expected to improve individualised pregnancy management through the better understanding of genetic aetiology of congenital anomalies caused by variants in a single gene.

This paper explores the cost-effectiveness of identifying an additional abnormality, prenatally, by including trio ES to currently applied methods, including QF-PCR and CMA.

## Methods

A model-based economic analysis was conducted alongside the Prenatal Assessment of Genomes and Exomes (PAGE) Study in order to assess the cost-effectiveness of 4 prenatal genetic test strategies. Our analysis was undertaken from a healthcare perspective. The outcome was measured as cost per additional genetic diagnosis, where it was assumed that each genetic diagnosis was equivalent to the identification of a congenital structural abnormality. A decision tree model was utilised given the short time frame of the study. The model structure was informed by experts from the Birmingham Women's Hospital. The model was populated using patient level data (*n* = 298) from the prospective clinical study. The model was built and analysed using TreeAge Pro 2018 [13].

### PAGE Study Recruitment

The PAGE study aimed to determine a cost-effective genome sequencing assay for improved prenatal genetic diagnosis and individualised pregnancy management. Methodological details of the recruitment process for PAGE have been published elsewhere [14]. Briefly, prospective women were counselled for recruitment into the PAGE study if they were attending a fetal medicine clinic for clinically indicated invasive diagnostic procedures. Women were included if they met the following inclusion criteria: (1) undergone prenatal invasive testing following the identification of one or more anomalies; (2) received a normal result for aneuploidy following a QF-PCR, conventional karyotyping (G-banding) or non-invasive prenatal test; (3) received consent to the PAGE study from the partner of the prospective woman.

### Model and Diagnostic Strategies

Four diagnostic strategies were examined for the purpose of this model, as follows:

*Strategy 1*. A CMA test was undertaken following a negative QF-PCR result. A positive result was indicative of a pathogenic finding; a negative finding indicated a normal result.

*Strategy 2*. An ES test was undertaken following a negative QF-PCR result. A positive ES was indicative of a pathogenic finding, whilst a negative finding indicated a normal result.

*Strategy 3*. A CMA test was undertaken following a negative QF-PCR result. If the CMA result was negative, then ES testing was undertaken. This stepwise approach was the “as per protocol” strategy in the PAGE study. The Evaluation of Array Comparative genomic Hybridisation (EACH) study noted that CMA had a “sample receipt to report” duration of 15 days (IQR 12–25) [9]. Recent data from the Baylor College in Texas have noted that for ES, an average “turnaround time” (TAT) for initial reporting (excluding tissue culture time) is 14 days [15]. This model assumed the TAT for CMA was 14 days and the TAT for ES was an additional 21 days. This implies that the TAT for the stepwise was 35 days. Therefore, when comparing the stepwise pathway to ES alone, the former had a slower TAT.

*Strategy 4*. A CMA and ES tests were undertaken at the same time following a negative QF-PCR result. CMA results were considered likely to be available before the ES results.

The model structure is presented in online supplementary Figure S1 (see www.karger.com/doi/10.1159/000504976 for all online suppl. material). A CMA and ES tests were undertaken for all patients included in the model. For strategies involving only 1 test, patients were reallocated to branches as if the missing test was negative. The test results of the patients also defined the distribution used to derive the branch probabilities. Note that no separate parameters are needed for test results in strategies 1 and 2. For example, the probability of a positive CMA test in strategy 1 is necessarily equal to the overall probability of a positive CMA test in strategies 3 and 4.

### Costs and Resource Data

Resource costs were divided into 2 categories: (1) costs incurred from genetic testing; (2) costs incurred from other medical management. Costs associated with genetic testing included CMA and ES. The cost of CMA was GBP 345, which included DNA extraction. This was obtained from West Midlands Regional Genetics Laboratory. The cost of ES was GBP 2,100, which included the cost of sequencing trio exome samples. This value was an average of costing figures obtained from 2 sources: (1) West Midlands Regional Genetics Laboratory, Birmingham Women's Hospital, UK (GBP 2,500); (2) Department of Genetics, Guy's and St. Thomas' NHS Foundation Trust, UK (GBP 1,700). The costs for genetic testing included staffing, consumables, administration, capital, bioinformatics, storage, reporting, and clinical review panel meetings.

Costs associated with medical management included pregnancy loss (termination of pregnancy [TOP] and spontaneous loss), delivery mode (vaginal delivery [VD], elective caesarean, and emergency caesarean), follow-up, and post-partum care costs. TOP costs were obtained from the NHS National Tariffs [16]. Costs associated with mode of delivery were obtained from the Personal Social Services Research Unit (PSSRU) National Tariff Payment System and the West Midlands Fetal Medicine Centre, Birmingham Women's Hospital, UK [17]. Follow-up costs were only included for those with a positive diagnostic result, as further counselling was required. This included 245 min consultations with a geneticist, and four 1-h consultations with a specialist midwife. Staffing costs were calculated using the “unit costs for hospital-based doctors” from the PSSRU [18]. Costs associated with post-partum care were provided by the Neonatal Unit, Birmingham Women's Hospital, UK [19]. A gamma distribution for cost parameters was fitted to the model (Table 1). All costs are presented in UK Sterling for 2016–2017. See online supplementary Table S1 for additional information.

### Outcome Measure

The outcome in this model was measured as one additional genetic diagnosis. This is reported in terms of the incremental cost-effectiveness ratio (ICER), which indicates the additional cost per additional genetic diagnosis.

### Analyses

Two key analyses were undertaken. *Analysis 1*(A1) examined all missing and complete case data (missing data included pregnancy outcomes [delivery mode: 13%; live births: 3% (VD or caesarean type was not specified); overall: approximately 16%] and test results [VUS: 17%]). *Analysis 2*(A2) analysed only the complete cases. An additional exploratory analysis was undertaken for each phenotype group (brain, skeletal/limb/spinal, cardiac, abdominal/gastro, nuchal translucency, single anomaly and multiple anomalies). The results report findings for single and multiple anomalies only. VUS cases were excluded from the sub-analysis, as the exact anomaly identified was unknown.

### Assumptions

Assumptions were applied to increase the reliability and validity of the results. The missing data in A1 included both delivery mode (16% missing) and test failure (17% missing). It was therefore assumed that missing data were proportionate to the complete cases within the dataset. This assumption ensured valid samples were not excluded unnecessarily. Furthermore, intrauterine demise, miscarriages, and stillbirths were assumed to be TOP cases, as the fetus had died in utero. Additionally, VUS cases that were not validated at PAGE clinical review panel meetings were assumed to be negative ES cases, whilst VUS cases that were validated were assumed to be positive ES cases (as these were considered to be clinically relevant). ES costs were also assumed to be an average of cost data from 2 centres (Table 1). The cost data (Table 1) were converted and presented in EUR and USD from UK sterling using 2017 conversion rates [20] to provide a wider perspective and generalisability of findings. However, doing so assumed all costing figures to be of equal value across countries, despite being obtained for a UK setting.

### Sensitivity Analysis

A limited deterministic sensitivity analysis (DSA) was performed alongside the analysis. A DSA tests the uncertainty surrounding the model parameters by varying the value of one parameter whilst maintaining the mean values for all other parameters. This means that any diagnostic strategy can become the preferred option should one parameter be highly sensitive. The following changes were made: (1) The cost of ES was assumed to decrease by 50% over time, and the ICERs were assessed at 10% decrements. (2) In A1, one scenario assumed all forms of unrecorded delivery were TOP (as a sample review indicated from our annual report that this was the most likely associated outcome [the centre was just not informed]). (3) In A1, a separate scenario assumed all forms of unrecorded delivery were VD. These choices reflect the fact that it is impossible to predict the pregnancy outcome due to the variety of anomalies and the differences in prognosis for a fetus.

A probabilistic sensitivity analysis (PSA) was undertaken to determine the level of uncertainty within the model. A Dirichlet distribution was used for all probabilities of each possible CMA and ES result. The test sensitivities and specificities were inferred from the sampled probability sets. A Dirichlet distribution for pregnancy outcome parameters was also fitted to the model. Table 2 presents the model parameters used within *A1*. The parameters for *A2* and the subgroup analysis are presented in online supplementary Table S2–9. Cost-effectiveness values were derived by simultaneously selecting random values from each distribution. 10,000 iterations were produced to provide an indication of how the variation in the test sensitivity and specificity affects the possible values of the ICER. A cost-effectiveness acceptability curve (CEAC) was then produced to examine the probability of cost-effectiveness per test strategy at a given willingness to pay (WTP). In this model, the WTP ranged between GBP 0 and GBP 50,000 per additional genetic diagnosis.

## Results

The results for A1 are presented in Table 3 and are explored within this section of the paper. The results for A2 have been presented and explained in the online suppl. Table S10, 11. In A1, CMA alone (strategy 1) was the least costly strategy, with a mean of GBP 3,654 (EUR 4,188/USD 4,687) per additional genetic diagnosis. 12.08% of all cases were identified to have an anomaly. CMA alone identified approximately 30% of all positive cases. ES alone (strategy 2) was more expensive but detected approximately 78% of all positive cases (8% of which would be solved by CMA alone). Combining the differences in overall costs and effects, ES alone costs an additional GBP 31,410 (EUR 36,001/USD 40,289) per additional genetic diagnosis compared to CMA alone.

**Fig. 1:**
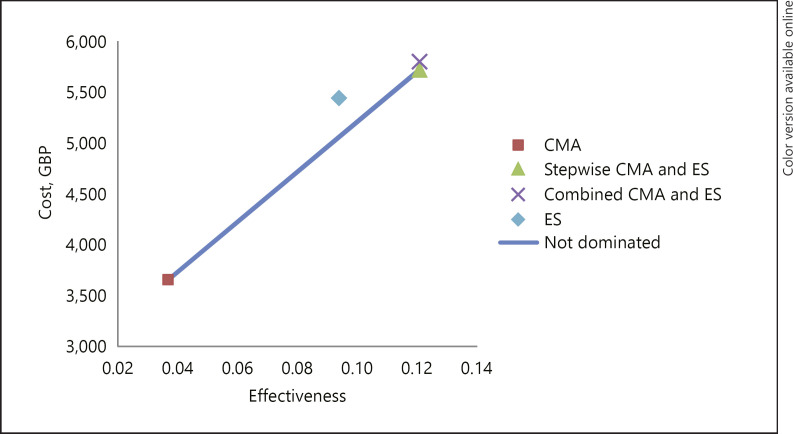
A1: Cost-effectiveness analysis, base case.

The stepwise (strategy 3) was more effective and more costly than the previous 2 strategies. This is because both tests were undertaken on most cases, which enabled it to identify all cases with a detectable anomaly. Even so, the additional cost per additional genetic diagnosis was GBP 24,657 (EUR 28,261/USD 31,627) when compared to CMA alone. This was lower than ES alone when compared to CMA alone (GBP 31,410 >GBP 24,657 [EUR 36,001 >EUR 28,261; USD 40,289 >USD 31,627]), meaning the stepwise dominated both strategies. This can be seen graphically in Figure 1 and numerically in the online supplementary Table S12 and 13. The combined (strategy 4) was dominated by the stepwise, as it was more costly with the same effectiveness (Table 3). These results suggest that CMA is the least costly strategy, but, if the WTP is at least GBP 24,657 (EUR 28,261/USD 31,627) per additional genetic diagnosis, the stepwise strategy would be preferred.

### Deterministic Sensitivity Analyses

Five additional scenarios were investigated following the base case analysis in A1 (Table 3). Each scenario tested a reduction in the cost of ES of up to 50% by 10% decrements. In each scenario, the pattern of dominance remained consistent with the base case analysis. An additional analysis was therefore undertaken to determine the point ES alone became a non-dominated strategy in A1. When the cost of ES is reduced to GBP 966 (EUR 1,107/USD 1,239; 54% reduction) in A1, the ICER for ES alone (GBP 11,532 [EUR 13,217/USD 14,792]) becomes lower than the stepwise (GBP 11,639 [EUR 13,340/USD 14,929]; Table 4). Therefore, ES alone is no longer dominated. A change in the assumptions placed on missing pregnancy outcome data was also evaluated. The pattern of dominance remained the same as the base case analysis when missing data were assumed to be either VD or TOP. See Table 4 for further details.

### Probabilistic Sensitivity Analysis

**Fig. 2:**
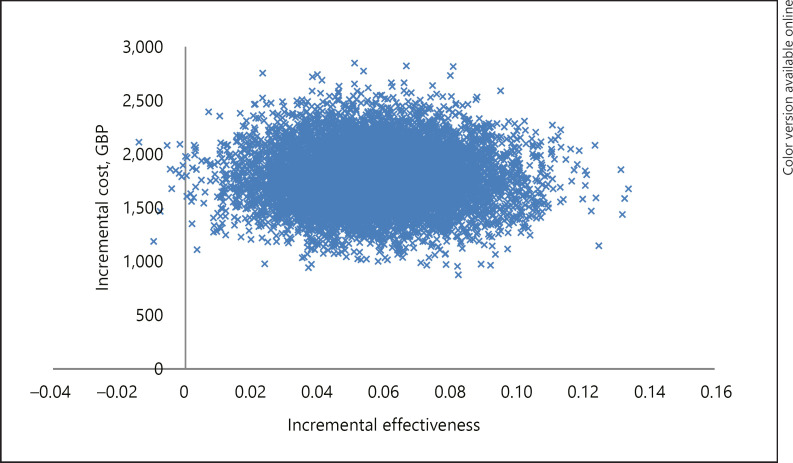
A1: Incremental cost-effectiveness plane: CMA and ES.

The PSA was undertaken to obtain the differences in the costs and effectiveness between each strategy in order to produce an incremental cost-effectiveness plane. Figure 2 shows the mean incremental costs and incremental effectiveness between CMA alone and ES alone for A1. The graph shows a large amount of parameter uncertainty, as the mean incremental costs and incremental effectiveness fall in the north east and the north-west quadrant of the plane. This indicates that ES is certain to be more costly than CMA, but there is a small probability, consistent with the data available, that ES is also less effective than CMA.

**Fig. 3:**
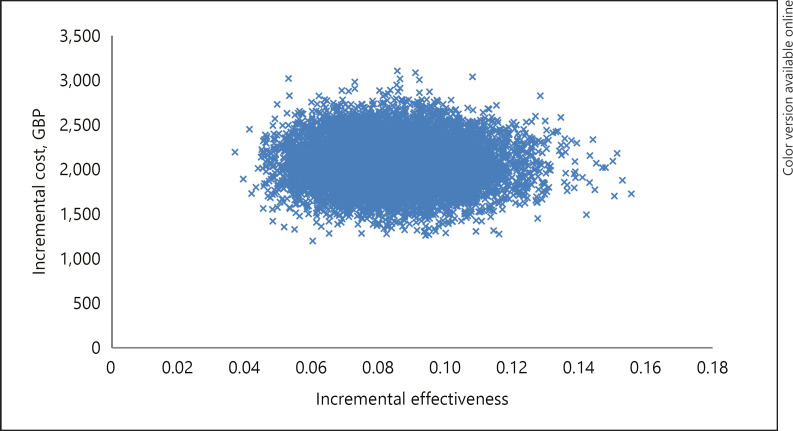
A1: Incremental cost-effectiveness plane: CMA and stepwise.

Figure 3 shows the mean incremental costs and incremental effectiveness between CMA alone and the stepwise. The graph shows some parameter uncertainty, although all points fall within the north east region of the plane. This implies that in all cases the stepwise will identify more abnormalities than CMA alone, but at an additional cost. Online supplementary Figure S2 shows the mean incremental costs and incremental effectiveness between ES alone and the stepwise. There is a large amount of parameter uncertainty despite all points falling within the north east region of the plane. This implies that in all cases the stepwise will identify more abnormalities than ES alone, but at an additional cost.

### Cost-Effective Acceptability Curves

**Fig. 4:**
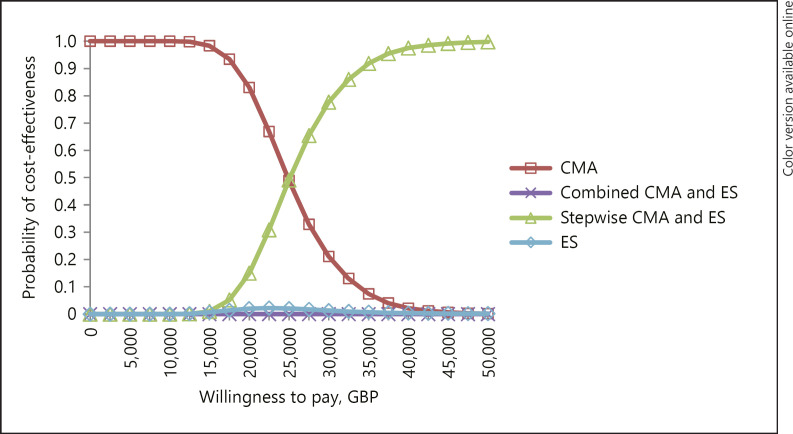
A1: Cost-effectiveness acceptability curve.

Figure 4 presents the CEAC for all strategies in A1 when ES is priced at GBP 2,100 (EUR 2,407/USD 2,694). For any possible WTP, the height of each curve shows the proportion of model replications at which the relevant strategy is cost-effective. The CEAC shows that ES alone is very unlikely to be cost-effective at any WTP, while the combined is never cost-effective. At a WTP of GBP 30,000 (EUR 34,385/USD 38,481), the probability that the stepwise is cost-effective is 78% and the probability that CMA alone is cost-effective is 21%. At a WTP of GBP 20,000 (EUR 22,923/USD 25,654), the probability that the stepwise is cost-effective is 15%, and the probability that CMA alone is cost-effective is 83%.

### Sub-Analysis

The testing strategies identified between 4 and 19% of all single and multiple anomalies identified by USS (online suppl. Table S14). The ICERs associated with ES alone exceeded the ICERs associated with the stepwise, when compared to CMA alone for both subgroups. The stepwise was therefore the optimal strategy. The ICERs associated with the stepwise for each subgroup differed by approximately GBP 20,000 (EUR 22,923/USD 25,654), due to the variation in the incremental cost and effectiveness. The analysis indicated a greater diagnostic yield (approximately 7%) and lower incremental cost (GBP 58) associated with the multiple anomaly subgroup compared to the single anomaly subgroup. It is therefore more cost-effective to undertake the stepwise when the USS is indicative of multiple anomalies.

A PSA was undertaken to derive the CEACs for both subgroups, which are presented in online supplementary Figure S10 and 11. *Multiple anomalies:* At WTP thresholds of GBP 20,000 (EUR 22,923/USD 25,654) and GBP 30,000 (EUR 34,385/USD 38,481), the probability that the stepwise is cost-effective exceeds the probability that either CMA alone or ES alone are cost-effective. *Single anomaly:* At WTP thresholds of GBP 20,000 (EUR 22,923/USD 25,654) and GBP 30,000 (EUR 34,385/USD 38,481), the probability that CMA alone is cost-effective exceeds the probability that either the stepwise or ES alone is cost-effective. The probability that the stepwise is more cost-effective compared to either the CMA alone or ES alone requires a WTP of at least GBP 35,000 (EUR 40,115/USD 44,894). Additional details of the analysis are presented in online supplementary Table S15. Online supplementary Tables S14 and 15 also present the findings for the additional subgroups analysed.

## Discussion

### Main Findings

This paper was built on a study conducted by Hillman et al. [8] that explored the cost-effectiveness of CMA against conventional prenatal testing techniques (e.g., QF-PCR). Comparing findings with Hillman et al. [8] was difficult, as the use of ES as a supplement for QF-PCR was not explored. Nevertheless, this paper demonstrates that prenatal ES alone identifies more abnormalities than CMA alone, despite being more costly. This means the diagnostic yield for ES alone is greater than CMA alone (A1: 78 >30%; A2: 82 >26%). From a health economics perspective, however, this model suggests ES alone is not good value for money when ES costs GBP 2,100 (EUR 2,407/USD 2,694) in both analyses. This is because the additional cost per additional fetal anomaly case genetic diagnosis for ES alone exceeds the cost for the stepwise (i.e., A1: GBP 31,410 >GBP 24,657 [EUR 36,001 >EUR 28,261; USD 40,289 >USD 31,627]). Therefore, in the case of A1, if the WTP exceeds GBP 31,410 (EUR 36,001/USD 40,289), the stepwise would still be recommended as its resolution is greater and its costs are lower. A similar conclusion was made in the sub-analysis for both groups.

ES alone was likely to be recommended when the cost of ES was reduced by 54% (A1), as it was no longer a dominated strategy. However, if the WTP were equal to the additional cost per additional case solved for the stepwise, the stepwise would be preferred. The model suggests that solving cases with multiple anomalies would likely be more cost-effective compared to solving for cases with single anomalies. This is due to the higher diagnostic yield and lower diagnosis cost of ES when solving for multiple anomalies. Even so, in each of the above scenarios, the direct effect of a test result on a pregnancy outcome was not evaluated; therefore, costs outside of a study setting might differ.

If the value of an additional case solved were equal to the value of an additional QALY (as defined by the National Institute for Health and Care Excellence [NICE]) the WTP would fall between GBP 20,000 (EUR 22,923/USD 25,654; lower threshold) and GBP 30,000 (EUR 34,385/USD 38,481; upper threshold). This model suggests that in the base case analysis neither the stepwise nor ES alone would be recommended at the lower threshold value, as the additional cost per genetic diagnosis for both strategies exceed GBP 20,000 (EUR 22,923/USD 25,654). At GBP 30,000 (EUR 34,385/USD 38,481) the stepwise would likely be recommended, as the additional cost per additional genetic diagnosis falls below the threshold. The model suggests the stepwise would remain the most cost-effective strategy at both thresholds when ES is reduced either 54%. If multiple anomalies were identified by USS, undertaking the stepwise would likely be the most cost-effective at either the GBP 20,000 (EUR 22,923/USD 25,654) or GBP 30,000 (EUR 34,385/USD 38,481) threshold, whereas using ES or stepwise for single anomalies was not cost-effective at either threshold. Even so, the sample size for the multiple anomaly subgroup was significantly lower compared to the single anomaly subgroup, which might have subsequently affected the results.

### Interpretation

The recent literature recommends CMA for identifying additional anomalies prenatally compared to techniques such as karyotype and QF-PCR, which are still used as standard care [5, 7, 21, 22]. This paper suggests that ES should also be considered in the testing process, as it was able to identify additional anomalies microarray was unable to solve. This is especially true for cases with multiple anomalies detected by USS, as the model suggests the incremental diagnostic yield is greater and incremental costs are lower compared to cases with single anomalies detected by USS. It is recommended that further research be undertaken to investigate the efficacy and cost-effectiveness of including ES as a supplement for CMA (stepwise). This will determine whether findings are consistent, and further support the case for implementing ES outside of clinical trial settings [5].

### Strengths and Limitations

A strength associated with this paper is the use of primary data. This provided greater validity to the analysis, as the data were collected specifically for the purpose of the study. This allowed for inclusion and exclusion criteria to be applied throughout the data collection process [13], which enabled the model to present a close depiction of reality. This paper also analysed the cost-effectiveness of ES per phenotype. To the best of our knowledge, this has not been done before.

There were few caveats to the study. First, in A1, missing data were assumed to be proportionate to the complete cases. “Live births” were also assumed to be VDs, although the delivery mode was not specific (i.e., VD, elective caesarean, or emergency caesarean). These assumptions may be incorrect, as it is impossible to predict or assume the delivery mode of a pregnancy. The study design and ethics approval of the PAGE study prevented the disclosure of test results until after pregnancy, meaning pregnancy outcomes were independent of the results. In reality, however, the test diagnosis would be known and would likely influence the pregnancy outcome. Therefore, the additional cost per genetic diagnosis may have been under-/overestimated. Nonetheless, delivery mode costs remained in the model to provide an estimate of the possible costs incurred to healthcare providers, along with an estimate of possible cost-effective strategies. The model evaluated an intermediate outcome, meaning the additional costs and effects incurred following the birth of a child with an anomaly solved, were not explored. Consequently, the additional cost per case solved may have been under-/overestimated.

The model assumed a validated VUS was potentially indicative of a pathogenic and clinically relevant finding, and a non-validated VUS was unlikely to be indicative of a clinically relevant finding. This assumption may be incorrect given the uncertainty associated with a VUS finding. This implies that cases assumed to be clinically relevant may not be and vice versa; therefore, affecting the ICERs derived. Even so, reporting a VUS result to parents might be inappropriate [5]. Therefore, excluding these cases from the model could have improved its validity. Hillman et al. [8] suggest an effectiveness score be computed when modelling VUS cases, but do not discuss computation methods. Given that ES has yet become a fully established routine clinical diagnostic test, and UK guidance on how and what to report is not readily available, the inclusion of VUS cases in this model is further complicated.

The model was unable to explore or quantify the possible loss/gain of utility to parents once a positive or VUS result was reported. The use of QALYs as an outcome measure was deemed unsuitable. This was due to the difficulty associated with counting QALYs when TOP is a possible outcome. TOP is the only alternative to delivering a child, once a positive or VUS is reported, as treatment following a pathogenic finding is generally not possible [6]. This supports the argument against the use of a QALY. Similar arguments apply to other measures such as the DALY.

Another limitation was determining whether a strategy was cost-effective. There are no set guidelines defining the monetary value per genetic diagnosis. To some extent, this paper assumed the value of a genetic diagnosis to be equivalent to that of a QALY. Using QALY thresholds to determine whether a strategy is cost-effective might have therefore been inappropriate. Defined guidelines would improve guidance on whether a test strategy should be considered. Additionally, the results were reported in EUR and USD. Doing so might have only provided a numerical estimation for countries using either currency, as the healthcare costs are likely to differ between countries. Finally, the model analysed 298 cases, of which only 11 were indicative of being CMA positive. The expectation, following discussions with experts in the field, was to identify a positive relationship between CMA positive and TOP cases. This was not observed. The sample size might have been too small to depict reality. It is recommended that further analyses be undertaken on a larger sample size to ensure findings are consistent.

To conclude, this model suggests that the stepwise is the most cost-effective strategy at a WTP of at least GBP 24,657 (EUR 28,261/USD 31,627). Implementing ES alone is only considered when the cost of ES was reduced by at least 50%. The findings also suggest that it is more cost-effective to analyse cases with multiple anomalies identified by USS compared to cases with single anomalies identified by USS. Even so, results should be treated with caution as the direct effects of test results on pregnancy outcomes were not modelled due to ethical reasons. Therefore, the additional cost per case solved might have been over-/underestimated. It is suggested that further research be undertaken to investigate this limitation, and that a larger sample size be used to improve the validity and reliability of findings.

## Statement of Ethics

Patients involved in the study had provided written consent. The study protocol was also approved by the institute's committee on human research.

## Disclosure Statement

The authors have no conflicts of interest to disclose.

## Funding Sources

The PAGE study is supported by a Health Innovation Challenge Fund Wellcome Trust (HICF-R7-396).

## Author Contributions

Shahela S. Kodabuckus was the health economist working on the project and, with Dr. Pelham M. Barton, was responsible for the health economic evaluation of the data. She also analysed the data and co-wrote the manuscript. Elizabeth Quinlan-Jones was the research midwife on the PAGE study. She was responsible for the day-to-day acquisition of exome sequencing data from clinical subjects. She also edited and added to the paper. Dr. Dominic J. McMullan, Prof. Eamonn R. Maher, Dr. Matthew E. Hurles, and Prof. Mark Kilby were investigators on the PAGE study. They jointly conceived the idea for the project and obtained funding form the Wellcome Trust. They had significant input into the design of the health economic analysis and its relation to the PAGE study. They co-wrote and edited the manuscript. Dr. Pelham M. Barton is a senior health economist at the University of Birmingham. He was the principle health economist overseeing the supervision of Shahela S. Kodabuckus. He had intellectual input into the health economic study design and co-wrote and edited the manuscript.

## Supplementary Material

Supplementary data

## Figures and Tables

**Table 1:** Model parameters for costs

Parameter	Mean	α	β	Source
*Costs, GBP*^†^				
Follow-up cost during pregnancy	646	100	0.1548	PSSRU (2016)
Vaginal delivery	1,775	100	0.0570	PSSRU (2016)
Elective caesarean section	1,775	100	0.0570	PSSRU (2016)
Emergency caesarean section	2,582	100	0.0387	PSSRU (2016)
Termination of pregnancy	730	100	0.1371	NHS national tariffs (2017)
Post-partum care	2,810	100	0.0356	Birmingham Women's Hospital (2017)
CMA	345	100	0.2899	Birmingham Women's Hospital (2017)
ES	2,100	100	0.0476	Birmingham Women's Hospital; Guy's and St. Thomas' NHS Foundation Trust (2017)

CMA, chromosomal microarray; ES, exome sequencing.† Gamma distribution has been fitted for parameter.

**Table 2:** A1: model parameters

Parameter	Mean	α	*n-*α	Source
Combined distribution*				
CMA positive + ES positive	0.0101	3	295	Study data
CMA positive + ES negative	0.0268	8	290	Study data
CMA negative + ES negative	0.8792	262	36	Study data
CMA negative + ES positive	0.0839	25	273	Study data

Pregnancy outcome after positive CMA*				
Termination of pregnancy	0.4545	5	6	Study data
Vaginal delivery	0.0909	1	10	Study data
Emergency caesarean section	0.0909	1	10	Study data
Elective caesarean section	0.3636	4	7	Study data

Pregnancy outcome after negative CMA*				
Termination of pregnancy	0.3554	102	185	Study data
Vaginal delivery	0.3972	114	173	Study data
Emergency caesarean section	0.1185	34	253	Study data
Elective caesarean section	0.1289	37	250	Study data

Pregnancy outcome after positive ES*				
Termination of pregnancy	0.5714	16	12	Study data
Vaginal delivery	0.1786	5	23	Study data
Emergency caesarean section	0.0714	2	26	Study data
Elective caesarean section	0.1786	5	23	Study data

Pregnancy outcome after negative ES*				
Termination of pregnancy	0.3370	91	179	Study data
Vaginal delivery	0.4074	110	160	Study data
Emergency caesarean section	0.1222	33	237	Study data
Elective caesarean section	0.1333	36	234	Study data

A beta distribution is a family of continuous probability distributions defined on the interval (0, 1), denoted by α and β, where α is the number of successes in a trial and β the number of failures. A positive CMA or ES result was indicative of an abnormal diagnosis. A negative CMA or ES result was indicative of normal diagnosis, meaning an anomaly was not identified in the fetus. Approximately 12% of all cases were detected to have an abnormal diagnosis. Outcome rates were based on cases post-delivery, meaning the test results were communicated to the parents at this point. MD, missing data accounted for (analysis one); CMA, chromosomal microarray; ES, exome sequencing. (* Dirichlet distribution is a multivariate generalisation of beta distribution.)

**Table 3:** A1: incremental ICERs for the base case and 5 scenario analyses

Strategy	Cost, GBP		Effectiveness		ICER
	mean	incremental	mean	incremental	
Base case					
*CMA alone (strategy 1)*	3,654		0.0369		
*ES alone (strategy 2)*	5,446	1,792	0.0940	0.0570	31,410
*CMA then ES (strategy 3)*	5,723	2,069	0.1208	0.0839	24,657
*CMA and ES (strategy 4)*	5,800	2,146	0.1208	0.0839	25,581

Scenario 1					
*CMA alone (strategy 1)*	3,654		0.0369		
*ES alone (strategy 2)*	5,236	1,582	0.0940	0.0570	27,729
*CMA then ES (strategy 3)*	5,520	1,866	0.1208	0.0839	22,246
*CMA and ES (strategy 4)*	5,590	1,936	0.1208	0.0839	23,078

Scenario 2					
*CMA alone (strategy 1)*	3,654		0.0369		
*ES alone (strategy 2)*	5,026	1,372	0.0940	0.0570	24,048
*CMA then ES (strategy 3)*	5,318	1,664	0.1208	0.0839	19,836
*CMA and ES (strategy 4)*	5,380	1,726	0.1208	0.0839	20,575

Scenario 3					
*CMA alone (strategy 1)*	3,654		0.0369		
*ES alone (strategy 2)*	4,816	1,162	0.0940	0.0570	20,367
*CMA then ES (strategy 3)*	5,116	1,462	0.1208	0.0839	17,425
*CMA and ES (strategy 4)*	5,170	1,516	0.1208	0.0839	18,072

Scenario 4					
*CMA alone (strategy 1)*	3,654		0.0369		
*ES alone (strategy 2)*	4,606	952	0.0940	0.0570	16,685
*CMA then ES (strategy 3)*	4,914	1,260	0.1208	0.0839	15,014
*CMA and ES (strategy 4)*	4,960	1,306	0.1208	0.0839	15,568

Scenario 5					
*CMA alone (strategy 1)*	3,654		0.0369		
*ES alone (strategy 2)*	4,396	742	0.0940	0.0570	13,004
*CMA then ES (strategy 3)*	4,711	1,057	0.1208	0.0839	12,603
*CMA and ES (strategy 4)*	4,750	1,096	0.1208	0.0839	13,065

Base case: assume ES is GBP 2,100. Scenario 1: Assume ES has decreased by 10% and is therefore GBP 1,890. Scenario 2: Assume ES has decreased by 20% and is therefore GBP 1,680. Scenario 3: Assume ES has decreased by 30% and is therefore GBP 1,470. Scenario 4: Assume ES has decreased by 40% and is therefore GBP 1,260. Scenario 5: Assume ES has decreased by 50% and is therefore GBP 1,050. CMA, chromosomal microarray; ES, exome sequencing.

**Table 4:** A1: Incremental ICERs for the deterministic analyses

Strategy	Cost, GBP		Effectiveness		ICER
	mean	incremental	mean	incremental	
Cost of ES is GBP 966 (54% reduction in cost)					
CMA alone (strategy 1)	3,654		0.0369		
ES alone (strategy 2)	4,312	0.0940	0.0570	11,532
CMA then ES (strategy 3)	4,630	976	0.1208	0.0839	11,639
CMA and ES (strategy 4)	4,666	658	1,012	0.1208	0.0839	12,064

Missing outcomes assumed to be VD			
CMA alone (strategy 1)	3,830		0.0369		
ES alone (strategy 2)	5,622	1,792	0.0940	0.0570	31,410
CMA then ES (strategy 3)	5,894	2,063	0.1208	0.0839	24,595
CMA and ES (strategy 4)	5,971	2,141	0.1208	0.0839	25,519

Missing outcomes assumed to be TOP				
CMA alone (strategy 1)	3,328		0.0369		
ES alone (strategy 2)	5,120	1,792	0.0940	0.0570	31,410
CMA then ES (strategy 3)	5,394	2,065	0.1208	0.0839	24,619
CMA and ES (strategy 4)	5,471	2,143	0.1208	0.0839	25,543

CMA, chromosomal microarray; ES, exome sequencing; VD, vaginal delivery; TOP, termination of pregnancy.
