# Supplementary material for: Exome Sequencing for Prenatal Detection of Genetic Abnormalities in Fetal Ultrasound Anomalies: An Economic Evaluation
Source: Fetal Diagn Ther. 2020 Jan 21;47(7):554–64. doi: 10.1159/000504976 (PMC7446299; doi:10.1159/000504976)

Supplementary Materials

Table S1. ***Description of Costs***

Cost	Description
Termination of Pregnancy	The unit cost of a termination of pregnancy varied based on the number of weeks of gestation (£397 for under 14 weeks, £498 for 14-20 weeks; £620 for over 20+ weeks). Feticide costs (£500.93) were applied to every TOP case that exceeded 22 weeks gestation (not inclusive of stillbirths, Intrauterine Demise [IUD] and miscarriages). This was obtained from West Midlands Fetal Medicine Centre, Birmingham Women's Hospital, UK. The proportion of cases that fell into each category was multiplied by the unit cost for each weeks of gestation category. This derived an aggregated value of approximately £730.
Vaginal Delivery, Elective Caesarean and Emergency Caesarean	Vaginal Delivery and elective caesarean costs were £1775, indicating delivery without complications. The cost of emergency caesarean was £2582, as it was assumed that complications were encountered.
Follow-up	The unit cost for follow-up was £646, which assumed an hourly rate of £110 per hour for a specialist nurse (Band 6 cost for

	each hour of patient contact) and £137 per hour for a medical consultant.
Post-Partum Care	The cost of post-partum care varied depending on the unit the baby was admitted to and the amount of time spent. The unit cost per day for Intensive Care, High Dependency, Special Care and Transitional Care was £1076, £862, £409 and £373, respectively. The proportion of cases requiring post-partum care, relative to the overall sample, was multiplied by the average cost for all units (with a trimmed mean of 10%). An aggregate value of £2810 was derived.

Table S2. ***A2:*** **Model Parameters**

**Parameter**	**Mean**	**α**	**n -α**	**Source**
**Combined Distribution***				
CMA Positive + ES Positive	0.0120	3	247	Study Data
CMA Positive + ES Negative	0.0240	6	244	Study Data
CMA Negative + ES Negative	0.8640	216	34	Study Data
CMA Negative + ES Positive	0.1000	25	225	Study Data
**Pregnancy outcome after positive CMA***				
Termination of Pregnancy	0.5556	5	4	Study Data
Vaginal delivery	0.0000	0	9	Study Data
Emergency caesarean section	0.1111	1	8	Study Data
Elective caesarean section	0.3333	3	6	Study Data
**Pregnancy outcome after negative CMA***				
Termination of Pregnancy	0.3610	87	154	Study Data
Vaginal delivery	0.3942	95	146	Study Data
Emergency caesarean section	0.1162	28	213	Study Data
Elective caesarean section	0.1286	31	210	Study Data
**Pregnancy outcome after positive ES***				
Termination of Pregnancy	0.5714	16	12	Study Data
Vaginal delivery	0.1786	5	23	Study Data
Emergency caesarean section	0.0714	2	26	Study Data
Elective caesarean section	0.1786	5	23	Study Data
**Pregnancy outcome after negative ES***				
Termination of Pregnancy	0.3423	76	146	Study Data
Vaginal delivery	0.4054	90	132	Study Data
Emergency caesarean section	0.1216	27	195	Study Data
Elective caesarean section	0.1306	29	193	Study Data

A beta distribution is a family of continuous probability distributions defined on the interval [0,1], denoted by α and β, where α is the number of successes in a trial and β the number of failures.
**Dirichlet distribution is a multivariate generalisation of beta distribution*
CC: Complete Cases Only (Analysis two) | CMA: Chromosomal Microarray | ES: Exome Sequencing

Table S3. ***Phenotype Single:*** **Model Parameters**

Parameter	Mean	α	n-α	Source
**Combined Distribution***				
CMA Positive + ES Positive	0.0103	2	192	Study Data
CMA Negative + ES Positive	0.0619	12	182	Study Data
CMA Negative + ES Negative	0.8969	174	20	Study Data
CMA Positive + ES Negative	0.0309	6	188	Study Data
**Pregnancy outcome after positive CMA***				
Termination of Pregnancy	0.5000	4	4	Study Data
Vaginal delivery	0.0000	0	8	Study Data
Emergency caesarean section	0.1250	1	7	Study Data
Elective caesarean section	0.3750	3	5	Study Data
**Pregnancy outcome after negative CMA***				
Termination of Pregnancy	0.3333	62	124	Study Data
Vaginal delivery	0.4247	79	107	Study Data
Emergency caesarean section	0.1183	22	164	Study Data
Elective caesarean section	0.1237	23	163	Study Data
**Pregnancy outcome after positive ES***				
Termination of Pregnancy	0.6429	9	5	Study Data
Vaginal delivery	0.1429	2	12	Study Data
Emergency caesarean section	0.0000	0	14	Study Data
Elective caesarean section	0.2143	3	11	Study Data
**Pregnancy outcome after negative ES***				
Termination of Pregnancy	0.3167	57	123	Study Data
Vaginal delivery	0.4278	77	103	Study Data
Emergency caesarean section	0.1278	23	157	Study Data
Elective caesarean section	0.1278	23	157	Study Data

*A beta distribution is a family of continuous probability distributions defined on the interval [0,1], denoted by α and β, where α is the number of successes in a trial and β the number of failures. *Dirichlet distribution is a multivariate generalisation of beta distribution. CMA: Chromosomal Microarray | ES: Exome Sequencing*

Table S4. ***Phenotype Multiple:*** **Model Parameters**

Parameter	Mean	α	n-α	Source
**Combined Distribution***				
CMA Positive + ES Positive	0.0192	1	51	Study Data
CMA Negative + ES Positive	0.1346	7	45	Study Data
CMA Negative + ES Negative	0.8077	42	10	Study Data
CMA Positive + ES Negative	0.0385	2	50	Study Data
**Pregnancy outcome after positive CMA***				
Termination of Pregnancy	0.3333	1	2	Study Data
Vaginal delivery	0.3333	1	2	Study Data
Emergency caesarean section	0.0000	0	3	Study Data
Elective caesarean section	0.3333	1	2	Study Data
**Pregnancy outcome after negative CMA***				
Termination of Pregnancy	0.5306	26	23	Study Data
Vaginal delivery	0.2653	13	36	Study Data
Emergency caesarean section	0.0612	3	46	Study Data
Elective caesarean section	0.1429	7	42	Study Data
**Pregnancy outcome after positive ES***				
Termination of Pregnancy	0.6250	5	3	Study Data
Vaginal delivery	0.0000	0	8	Study Data
Emergency caesarean section	0.1250	1	7	Study Data
Elective caesarean section	0.2500	2	6	Study Data
**Pregnancy outcome after negative ES***				
Termination of Pregnancy	0.5000	22	22	Study Data
Vaginal delivery	0.3182	14	30	Study Data
Emergency caesarean section	0.0455	2	42	Study Data
Elective caesarean section	0.1364	6	38	Study Data

*A beta distribution is a family of continuous probability distributions defined on the interval [0,1], denoted by α and β, where α is the number of successes in a trial and β the number of failures. *Dirichlet distribution is a multivariate generalisation of beta distribution. CMA: Chromosomal Microarray | ES: Exome Sequencing*

Table S5. ***Phenotype Skeletal/Limb/Spinal:*** **Model Parameters**

Parameter	Mean	α	n-α	Source
**Combined Distribution***				
CMA Positive + ES Positive	0.0345	1	28	Study Data
CMA Negative + ES Positive	0.0690	2	27	Study Data
CMA Negative + ES Negative	0.8621	25	4	Study Data
CMA Positive + ES Negative	0.0345	1	28	Study Data
**Pregnancy outcome after positive CMA***				
Termination of Pregnancy	0.5000	1	1	Study Data
Vaginal delivery	0.0000	0	2	Study Data
Emergency caesarean section	0.0000	0	2	Study Data
Elective caesarean section	0.5000	1	1	Study Data
**Pregnancy outcome after negative CMA***				
Termination of Pregnancy	0.3333	9	18	Study Data
Vaginal delivery	0.3704	10	17	Study Data
Emergency caesarean section	0.0741	2	25	Study Data
Elective caesarean section	0.2222	6	21	Study Data
**Pregnancy outcome after positive ES***				
Termination of Pregnancy	1.0000	3	0	Study Data
Vaginal delivery	0.0000	0	3	Study Data
Emergency caesarean section	0.0000	0	3	Study Data
Elective caesarean section	0.0000	0	3	Study Data
**Pregnancy outcome after negative ES***				
Termination of Pregnancy	0.2692	7	19	Study Data
Vaginal delivery	0.3846	10	16	Study Data
Emergency caesarean section	0.0769	2	24	Study Data
Elective caesarean section	0.2692	7	19	Study Data

*A beta distribution is a family of continuous probability distributions defined on the interval [0,1], denoted by α and β, where α is the number of successes in a trial and β the number of failures. *Dirichlet distribution is a multivariate generalisation of beta distribution. CMA: Chromosomal Microarray | ES: Exome Sequencing*

Table S6. *Phenotype Cardiac:* Model Parameters

Parameter	Mean	α	n-α	Source
**Combined Distribution***				
CMA Positive + ES Positive	0.0000	0	43	Study Data
CMA Negative + ES Positive	0.1163	5	38	Study Data
CMA Negative + ES Negative	0.8372	36	7	Study Data
CMA Positive + ES Negative	0.0465	2	41	Study Data
**Pregnancy outcome after positive CMA***				
Termination of Pregnancy	0.0000	0	2	Study Data
Vaginal delivery	0.0000	0	2	Study Data
Emergency caesarean section	1.0000	2	0	Study Data
Elective caesarean section	0.0000	0	2	Study Data
**Pregnancy outcome after negative CMA***				
Termination of Pregnancy	0.3659	15	26	Study Data
Vaginal delivery	0.3659	15	26	Study Data
Emergency caesarean section	0.0976	4	37	Study Data
Elective caesarean section	0.1707	7	34	Study Data
**Pregnancy outcome after positive ES***				
Termination of Pregnancy	0.0000	0	5	Study Data
Vaginal delivery	0.4000	2	3	Study Data
Emergency caesarean section	0.0000	0	5	Study Data
Elective caesarean section	0.6000	3	2	Study Data
**Pregnancy outcome after negative ES***				
Termination of Pregnancy	0.3947	15	23	Study Data
Vaginal delivery	0.3421	13	25	Study Data
Emergency caesarean section	0.1053	4	34	Study Data
Elective caesarean section	0.1579	6	32	Study Data

*A beta distribution is a family of continuous probability distributions defined on the interval [0,1], denoted by α and β, where α is the number of successes in a trial and β the number of failures. *Dirichlet distribution is a multivariate generalisation of beta distribution. CMA: Chromosomal Microarray | ES: Exome Sequencing*

Table S7. ***Phenotype Abdominal/Gastro:*** **Model Parameters**

Parameter	Mean	α	n-α	Source
**Combined Distribution***				
CMA Positive + ES Positive	0.0000	0	19	Study Data
CMA Negative + ES Positive	0.0526	1	18	Study Data
CMA Negative + ES Negative	0.8421	16	3	Study Data
CMA Positive + ES Negative	0.1053	2	17	Study Data
**Pregnancy outcome after positive CMA***				
Termination of Pregnancy	0.5000	1	1	Study Data
Vaginal delivery	0.0000	0	2	Study Data
Emergency caesarean section	0.5000	1	1	Study Data
Elective caesarean section	0.0000	0	2	Study Data
**Pregnancy outcome after negative CMA***				
Termination of Pregnancy	0.5294	9	8	Study Data
Vaginal delivery	0.2941	5	12	Study Data
Emergency caesarean section	0.1176	2	15	Study Data
Elective caesarean section	0.0588	1	16	Study Data
**Pregnancy outcome after positive ES***				
Termination of Pregnancy	1.0000	1	0	Study Data
Vaginal delivery	0.0000	0	1	Study Data
Emergency caesarean section	0.0000	0	1	Study Data
Elective caesarean section	0.0000	0	1	Study Data
**Pregnancy outcome after negative ES***				
Termination of Pregnancy	0.5000	9	9	Study Data
Vaginal delivery	0.2778	5	13	Study Data
Emergency caesarean section	0.1667	3	15	Study Data
Elective caesarean section	0.0556	1	17	Study Data

*A beta distribution is a family of continuous probability distributions defined on the interval [0,1], denoted by α and β, where α is the number of successes in a trial and β the number of failures. *Dirichlet distribution is a multivariate generalisation of beta distribution. CMA: Chromosomal Microarray | ES: Exome Sequencing*

Table S8. ***Phenotype Nuchal Translucency:*** **Model Parameters**

Parameter	Mean	α	n-α	Source
**Combined Distribution***				
CMA Positive + ES Positive	0.0000	0	45	Study Data
CMA Negative + ES Positive	0.0889	4	41	Study Data
CMA Negative + ES Negative	0.8889	40	5	Study Data
CMA Positive + ES Negative	0.0222	1	44	Study Data
**Pregnancy outcome after positive CMA***				
Termination of Pregnancy	1.0000	1	0	Study Data
Vaginal delivery	0.0000	0	1	Study Data
Emergency caesarean section	0.0000	0	1	Study Data
Elective caesarean section	0.0000	0	1	Study Data
**Pregnancy outcome after negative CMA***				
Termination of Pregnancy	0.2045	9	35	Study Data
Vaginal delivery	0.5909	26	18	Study Data
Emergency caesarean section	0.1136	5	39	Study Data
Elective caesarean section	0.0909	4	40	Study Data
**Pregnancy outcome after positive ES***				
Termination of Pregnancy	1.0000	4	0	Study Data
Vaginal delivery	0.0000	0	4	Study Data
Emergency caesarean section	0.0000	0	4	Study Data
Elective caesarean section	0.0000	0	4	Study Data
**Pregnancy outcome after negative ES***				
Termination of Pregnancy	0.1463	6	35	Study Data
Vaginal delivery	0.6341	26	15	Study Data
Emergency caesarean section	0.1220	5	36	Study Data
Elective caesarean section	0.0976	4	37	Study Data

*A beta distribution is a family of continuous probability distributions defined on the interval [0,1], denoted by α and β, where α is the number of successes in a trial and β the number of failures. *Dirichlet distribution is a multivariate generalisation of beta distribution. CMA: Chromosomal Microarray | ES: Exome Sequencing*

Table S9. ***Phenotype Brain:*** **Model Parameters**

Parameter	Mean	α	n-α	Source
**Combined Distribution***				
CMA Positive + ES Positive	0.0385	1	25	Study Data
CMA Negative + ES Positive	0.0000	0	26	Study Data
CMA Negative + ES Negative	0.9615	25	1	Study Data
CMA Positive + ES Negative	0.0000	0	26	Study Data
**Pregnancy outcome after positive CMA***				
Termination of Pregnancy	1.0000	1	0	Study Data
Vaginal delivery	0.0000	0	1	Study Data
Emergency caesarean section	0.0000	0	1	Study Data
Elective caesarean section	0.0000	0	1	Study Data
**Pregnancy outcome after negative CMA***				
Termination of Pregnancy	0.5600	14	11	Study Data
Vaginal delivery	0.1200	3	22	Study Data
Emergency caesarean section	0.1600	4	21	Study Data
Elective caesarean section	0.1600	4	21	Study Data
**Pregnancy outcome after positive ES***				
Termination of Pregnancy	1.0000	1	0	Study Data
Vaginal delivery	0.0000	0	1	Study Data
Emergency caesarean section	0.0000	0	1	Study Data
Elective caesarean section	0.0000	0	1	Study Data
**Pregnancy outcome after negative ES***				
Termination of Pregnancy	0.5600	14	11	Study Data
Vaginal delivery	0.1200	3	22	Study Data
Emergency caesarean section	0.1600	4	21	Study Data
Elective caesarean section	0.1600	4	21	Study Data

*A beta distribution is a family of continuous probability distributions defined on the interval [0,1], denoted by α and β, where α is the number of successes in a trial and β the number of failures. *Dirichlet distribution is a multivariate generalisation of beta distribution. CMA: Chromosomal Microarray | ES: Exome Sequencing*

Table S10. Order of Dominance, *A1*

Strategy	Cost (£) Mean	Cost (£) Incremental	Effectiveness Mean	Effectiveness Incremental	ICER
**Base case**					
CMA alone *(Strategy One)*	3654	0	0.0369	0.0000	0
WES alone *(Strategy Two)*	5446	1792	0.0940	0.0570	31410
CMA then WES *(Strategy Three)*	5723	277	0.1208	0.0268	10307
CMA and WES *(Strategy Four)*	5800	78	0.1208	0.0000	0
**Scenario 1**					
CMA alone *(Strategy One)*	3654	0	0.0369	0.0000	0
WES alone *(Strategy Two)*	5236	1582	0.0940	0.0570	27729
CMA then WES *(Strategy Three)*	5520	284	0.1208	0.0268	10596
CMA and WES *(Strategy Four)*	5590	70	0.1208	0.0000	0
**Scenario 2**					
CMA alone *(Strategy One)*	3654	0	0.0369	0.0000	0
WES alone *(Strategy Two)*	5026	1372	0.0940	0.0570	24048
CMA then WES *(Strategy Three)*	5318	292	0.1208	0.0268	10885
CMA and WES *(Strategy Four)*	5380	62	0.1208	0.0000	0
**Scenario 3**					
CMA alone *(Strategy One)*	3654	0	0.0369	0.0000	0
WES alone *(Strategy Two)*	4816	1162	0.0940	0.0570	20367
CMA then WES *(Strategy Three)*	5116	300	0.1208	0.0268	11174
CMA and WES *(Strategy Four)*	5170	54	0.1208	0.0000	0
**Scenario 4**					
CMA alone *(Strategy One)*	3654	0	0.0369	0.0000	0
WES alone *(Strategy Two)*	4606	952	0.0940	0.0570	16685
CMA then WES *(Strategy Three)*	4914	308	0.1208	0.0268	11462
CMA and WES *(Strategy Four)*	4960	47	0.1208	0.0000	0
**Scenario 5**					
CMA alone *(Strategy One)*	3654	0	0.0369	0.0000	0
WES alone *(Strategy Two)*	4396	742	0.0940	0.0570	13004
CMA then WES *(Strategy Three)*	4711	315	0.1208	0.0268	11751
CMA and WES *(Strategy Four)*	4750	39	0.1208	0.0000	0

Base case: Assume WES is £2100

Scenario 1: Assume WES has decreased by 10% and is therefore £1890

Scenario 2: Assume WES has decreased by 20% and is therefore £1680

Scenario 3: Assume WES has decreased by 30% and is therefore £1470

Scenario 4: Assume WES has decreased by 40% and is therefore £1260

Scenario 5: Assume WES has decreased by 50% and is therefore £1050

CMA: Chromosomal Microarray | ES: Exome Sequencing

Table S11. ***A2 Analysis Results***

**Measure**	**Results**
**Detection Rates**	Table S12-S13 presents the findings for ***A2***. Briefly, 13.60% of all cases were identified to have an anomaly. CMA alone identified approximately 26% of all possible cases and ES alone identified approximately 82% of all possible cases. The stepwise and the combined strategies identified all possible cases.
**Incremental Cost Effectiveness Ratio (ICER)**	The findings presented in Table S12-S13 show that compared to the cost of CMA, ES alone was found to be the least costly strategy. Even so, the stepwise was able to identify all of the possible anomalies at an incremental cost of £266 (€305/$341), when compared to ES alone. The ICER derived for the stepwise is therefore lower than the ICER derived for ES alone. This implies that it is more cost-effective to employ the stepwise, as opposed to ES alone, in order to identify an additional genetic diagnosis. In this case, the dominant strategy is the stepwise. This can be seen graphically in Figure S3.
**Deterministic Sensitivity Analysis**	In the limited deterministic sensitivity analysis (DSA), five additional scenarios were investigated following the base case analysis in ***A2*** (Table S12-S13). Each scenario tested a reduction in the cost of ES of up to 50% by 10% decrements. The pattern of dominance remained consistent with the base case analysis in all but one scenario. When the cost of ES was reduced by 50% £1050[€1,203/$1,347]), the pattern of dominance changes, such that ES was no longer a dominated strategy. This is clear, as the ICER for ES alone is lower than the ICER for the stepwise. This therefore implies that, based on the values of the ICER's, ES alone can be considered as a suitable

	strategy. However, if the WTP threshold exceeds £10581(€12127/$13,572), the stepwise will still remain the preferred strategy. This is because the stepwise yields more, in terms of effectiveness.
**Incremental Cost Effectiveness Curves**	The PSA was undertaken to obtain the differences in the costs and effectiveness between each strategy in order to produce an incremental cost-effectiveness plane. Figure S5 shows the mean incremental costs and incremental effectiveness between CMA alone and ES alone for ***A2***. The graph shows a large amount of parameter uncertainty, as the mean incremental costs and incremental effectiveness falls in the north east and the north-west quadrant of the plane. This indicates that ES is certain to be more costly than CMA, but there is a small probability, consistent with the data available, that ES is also less effective than CMA.<br>Figure S6 shows the mean incremental costs and incremental effectiveness between CMA alone and the stepwise. The graph shows some parameter uncertainty, although all points fall within the north east region of the plane. This implies that in all cases the stepwise will identify more abnormalities than CMA alone, but at an additional cost. Figure S7 shows the mean incremental costs and incremental effectiveness between ES alone and the stepwise. There is a large amount of parameter uncertainty despite all points falling within the north east region of the plane. This implies that in all cases the stepwise will identify more abnormalities than ES alone, but at an additional cost.
**Cost Effectiveness Acceptability**	Figure S8 presents the CEAC for ***A2***, when the cost of ES is (€2,407/$2,694). At a WTP of £20,000 (€22,923/$25,654) the probability that the stepwise is cost-effective is approximately 36% and the probability that CMA alone is cost-effective is 56%. At a WTP of

**Curve**<br><br>**(CEAC)**	£30,000 (€34,385/$38,481) the probability that the stepwise cost-effective is 90% and the probability that CMA alone is cost-effective is 6%.<br><br>Figure S9 presents the CEAC for ***A2***, when the cost of ES is £1,050 (€ 1,203/$1,347). At a WTP of £20,000 (€22,923/$25,654) the probability that the stepwise is cost-effective is approximately 79% and the probability that CMA alone is cost-effective is less than 1%. At a WTP of £30,000 (€34,385/$38,481) the probability that the stepwise cost-effective is 94% and the probability that CMA alone is cost-effective is 0%.

Table S12. ***A2:*** **Incremental ICERs for the base case and five scenario analyses**

	**Cost (£)**		**Effectiveness**		
**Strategy**	**Mean**	**Incremental**	**Mean**	**Incremental**	**ICER**
**Base case**					
CMA alone (Strategy One)	3618		0.0360		
ES alone (Strategy Two)	5422	1804	0.1120	0.0760	23738
CMA then ES (Strategy Three)	5688	2070	0.1360	0.1000	20703
CMA and ES (Strategy Four)	5764	2146	0.1360	0.1000	21459
**Scenario 1**					
CMA alone (Strategy One)	3618		0.0360		
ES alone (Strategy Two)	5212	1594	0.1120	0.0760	20975
CMA then ES (Strategy Three)	5486	1868	0.1360	0.1000	18678
CMA and ES (Strategy Four)	5554	1936	0.1360	0.1000	19359
**Scenario 2**					
CMA alone (Strategy One)	3618		0.0360		
ES alone (Strategy Two)	5002	1384	0.1120	0.0760	18212
CMA then ES (Strategy Three)	5283	1665	0.1360	0.1000	16654
CMA and ES (Strategy Four)	5344	1726	0.1360	0.1000	17259
**Scenario 3**					
CMA alone (Strategy One)	3618		0.0360		
ES alone (Strategy Two)	4792	1174	0.1120	0.0760	15449
CMA then ES (Strategy Three)	5081	1463	0.1360	0.1000	14630
CMA and ES (Strategy Four)	5134	1516	0.1360	0.1000	15159
**Scenario 4**					
CMA alone (Strategy One)	3618		0.0360		
ES alone (Strategy Two)	4582	964	0.1120	0.0760	12685
CMA then ES (Strategy Three)	4879	1261	0.1360	0.1000	12605
CMA and ES (Strategy Four)	4924	1306	0.1360	0.1000	13059
**Scenario 5**					
CMA alone (Strategy One)	3618		0.0360		
ES alone (Strategy Two)	4372	754	0.1120	0.0760	9922
CMA then ES (Strategy Three)	4676	1058	0.1360	0.1000	10581
CMA and ES (Strategy Four)	4714	1096	0.1360	0.1000	10959

Base case: Assume ES is £2100
Scenario 1: Assume ES has decreased by 10% and is therefore £1890
Scenario 2: Assume ES has decreased by 20% and is therefore £1680
Scenario 3: Assume ES has decreased by 30% and is therefore £1470
Scenario 4: Assume ES has decreased by 40% and is therefore £1260
Scenario 5: Assume ES has decreased by 50% and is therefore £1050
CMA: Chromosomal Microarray | ES: Exome Sequencing

Table S13. Order of Dominance, *A2*

Strategy	Cost (£) Mean	Cost (£) Incremental	Effectiveness Mean	Effectiveness Incremental	ICER
**Base case**					
CMA alone *(Strategy One)*	3618	0	0.0360	0.0000	0
WES alone *(Strategy Two)*	5422	1804	0.1120	0.0760	23738
CMA then WES *(Strategy Three)*	5688	266	0.1360	0.0240	11091
CMA and WES *(Strategy Four)*	5764	76	0.1360	0.0000	0
**Scenario 1**					
CMA alone *(Strategy One)*	3618	0	0.0360	0.0000	0
WES alone *(Strategy Two)*	5212	1594	0.1120	0.0760	20975
CMA then WES *(Strategy Three)*	5486	274	0.1360	0.0240	11406
CMA and WES *(Strategy Four)*	5554	68	0.1360	0.0000	0
**Scenario 2**					
CMA alone *(Strategy One)*	3618	0	0.0360	0.0000	0
WES alone *(Strategy Two)*	5002	1384	0.1120	0.0760	18212
CMA then WES *(Strategy Three)*	5283	281	0.1360	0.0240	11721
CMA and WES *(Strategy Four)*	5344	60	0.1360	0.0000	0
**Scenario 3**					
CMA alone *(Strategy One)*	3618	0	0.0360	0.0000	0
WES alone *(Strategy Two)*	4792	1174	0.1120	0.0760	15449
CMA then WES *(Strategy Three)*	5081	289	0.1360	0.0240	12036
CMA and WES *(Strategy Four)*	5134	53	0.1360	0.0000	0
**Scenario 4**					
CMA alone *(Strategy One)*	3618	0	0.0360	0.0000	0
WES alone *(Strategy Two)*	4582	964	0.1120	0.0760	12685
CMA then WES *(Strategy Three)*	4879	296	0.1360	0.0240	12351
CMA and WES *(Strategy Four)*	4924	45	0.1360	0.0000	0
**Scenario 5**					
CMA alone *(Strategy One)*	3618	0	0.0360	0.0000	0
WES alone *(Strategy Two)*	4372	754	0.1120	0.0760	9922
CMA then WES *(Strategy Three)*	4676	304	0.1360	0.0240	12666
CMA and WES *(Strategy Four)*	4714	38	0.1360	0.0000	0

Base case: Assume WES is £2100

Scenario 1: Assume WES has decreased by 10% and is therefore £1890

Scenario 2: Assume WES has decreased by 20% and is therefore £1680

Scenario 3: Assume WES has decreased by 30% and is therefore £1470

Scenario 4: Assume WES has decreased by 40% and is therefore £1260

Scenario 5: Assume WES has decreased by 50% and is therefore £1050

CMA: Chromosomal Microarray | ES: Exome Sequencing

Table S14. ***Sub-group analyses***

Strategy	Cost (£) Mean	Cost (£) Incremental	Effectiveness Mean	Effectiveness Incremental	ICER
Phenotype: Multiple Anomalies					
CMA alone (Strategy One)	3659		0.0577		
ES alone (Strategy Two)	5428	1769	0.1538	0.0962	18399
CMA then ES (Strategy Three)	5668	2008	0.1923	0.1346	14920
CMA and ES (Strategy Four)	5789	2130	0.1923	0.1346	15820
Phenotype: Single Anomaly					
CMA alone (Strategy One)	3655		0.0412		
ES alone (Strategy Two)	5452	1797	0.0722	0.0309	58111
CMA then ES (Strategy Three)	5721	2066	0.1031	0.0619	33395
CMA and ES (Strategy Four)	5807	2152	0.1031	0.0619	34795
Phenotype: Skeletal/Limb/Spinal					
CMA alone (Strategy One)	3662		0.0690		
ES alone (Strategy Two)	5443	1781	0.1034	0.0345	51657
CMA then ES (Strategy Three)	5665	2003	0.1379	0.0690	29048
CMA and ES (Strategy Four)	5810	2148	0.1379	0.0690	31148
Phenotype: Cardiac					
CMA alone (Strategy One)	3656		0.0465		
ES alone (Strategy Two)	5439	1783	0.1163	0.0698	25555
CMA then ES (Strategy Three)	5695	2038	0.1628	0.1163	17528
CMA and ES (Strategy Four)	5792	2136	0.1628	0.1163	18368
Phenotype: Abdominal/Gastro					
CMA alone (Strategy One)	3671		0.1053		

ES alone (Strategy Two)	5458	1787	0.0526	-0.0526	-33961
CMA then ES (Strategy Three)	5600	1929	0.1579	0.0526	36655
CMA and ES (Strategy Four)	5821	2150	0.1579	0.0526	40855
Phenotype: Nuchal Translucency					
CMA alone (Strategy One)	3650		0.0222		
ES alone (Strategy Two)	5447	1797	0.0889	0.0667	26954
CMA then ES (Strategy Three)	5749	2099	0.1111	0.0889	23614
CMA and ES (Strategy Four)	5796	2146	0.1111	0.0889	24139
Phenotype: Brain					
CMA alone (Strategy One)	3654		0.0385		
ES alone (Strategy Two)	5462	1808	0.0385	0.0000	(undefined)
CMA then ES (Strategy Three)	5744	2090	0.0385	0.0000	(undefined)
CMA and ES (Strategy Four)	5825	2171	0.0385	0.0000	(undefined)

CMA: Chromosomal Microarray | ES: Exome Sequencing

Table S15. ***Sub-group Analysis Results***

Measure	Results
**Detection Rates**	The detection rates varied between strategies and subgroups (see Table S14). CMA detected between 2-10% of all phenotypes identified using USS. ES alone detected between 3-15% of all phenotypes identified using USS. The stepwise and the combined detected between 3-19% of all phenotypes identified using USS. It should be noted that all test strategies had a detection rate of approximately 3% for the brain subgroup. This is because both CMA and ES identified the same positive cases within the sample.
**Incremental Cost Effectiveness Ratio (ICER)**	Table S14 presents the ICER's for each subgroup. The ICER for ES alone and the combined approach exceeded the stepwise for all subgroups but the Brain. This means that compared to CMA, the stepwise is the dominant strategy. The results suggest that if the WTP is at least £36,900 (€42,290/$47,330), the stepwise will be the preferred option for all subgroups.<br>The findings associated to the Brain subgroup show the ICER's to be undefined. This is because there was no additional effectiveness per strategy, only additional costs. The findings therefore suggest that CMA will remain as the preferred strategy should the USS be indicative of an anomaly associated with the Brain, as CMA absolutely dominates all other strategies. Even so, the sample size for this subgroup was only 26. This might have been too small to show a true depiction of the cost-effectiveness of detecting an anomaly associated with the Brain.<br>The ICER's of each subgroup differed, meaning the necessary WTP for a strategy to be cost-effective varied. This was due to the different incremental costs and incremental effectiveness for each subgroup. It might therefore be more cost-effective to undertake the stepwise on

	selected subgroups, where the incremental costs are lower and the effectiveness is significantly greater. For example, cases with multiple anomalies detected by USS will likely be preferred over cases with a single anomaly detected, as the incremental effectiveness is greater and the incremental costs are lower, which in turn, produces a lower ICER.<br>Furthermore, if the WTP threshold is £30,000 (€34,385/$38,481), based on the ICERS alone, employing the stepwise would not be recommended if a single anomaly, abdominal, gastro, or brain related anomaly is identified by USS. This is because the ICER's for these sub-groups exceed the maximum threshold.
**Cost Effectiveness Acceptability Curve (CEAC)**	A PSA was undertaken to examine the level of uncertainty surrounding the parameters used for each phenotype subgroup. The PSA enabled the model to derive a CEAC for each group, which states the probability each strategy will be cost-effective.<br>*Phenotype: Multiple Anomalies*<br>Figure S10 presents the CEAC for the multiple anomaly subgroup. The figure shows that at a WTP of £20,000 (€22,923/$25,654), the probability that CMA is cost-effective is 29% and the probability that the stepwise is cost-effective is 56%. At a WTP of £30,000 (€34,385/$38,481), Figure S10 illustrates that the probability that CMA is cost-effective is 7%, the probability that ES alone is cost-effective is 10%, and the probability that stepwise is cost-effective is 83%.<br>*Phenotype: Single Anomaly*<br>Figure S11 presents the CEAC for the multiple anomaly subgroup. The figure shows that at a WTP of £20,000 (€22,923/$25,654), the probability that CMA is cost-effective is 95%, whereas the probability that stepwise is cost-effective is 4%. At a WTP of £30,000

	(€34,385/$38,481), the figure indicates that the probability that CMA is cost-effective is 64%, whereas the probability that stepwise is cost-effective is 35%. If the WTP is at least £35,000 (€40,115/$44,894), the probability that stepwise is cost-effective exceeds the probability that CMA is cost-effective. The findings suggest that the stepwise is likely to be more cost-effective when the USS is indicative of multiple anomalies compared to the single anomaly subgroup, as the probability is greater amongst the group. Nonetheless, the sample size of the multiple anomaly subgroup was much lower compared to the single anomaly subgroup. Therefore, the test results of the samples may have subsequently influenced the PSA findings.<br><br>*Phenotype: Skeletal/Limb/Spinal*<br>Figure S12 presents the CEAC for the subgroup associated with skeletal, limb and spinal anomalies. At a WTP of £20,000 (€22,923/$25,654) the probability that the stepwise is cost-effective is approximately 15% and the probability that CMA alone is cost-effective is 74%. At a WTP of £30,000 (€34,385/$38,481) the probability that the stepwise cost-effective is 32% and the probability that CMA alone is cost-effective is 56%. If the WTP is greater than £40,000 (€45,846 / $51,308), the probability that the stepwise is cost-effective will exceed the probability that CMA alone is cost-effective.<br>*Phenotype: Cardiac*<br>Figure S13 presents the CEAC for the subgroup associated with cardiac anomalies. At a WTP of £20,000 (€22,923/$25,654) the probability that the stepwise and CMA alone are cost-effective is approximately 45%. At a WTP of £30,000 (€34,385/$38,481) the

	probability that the stepwise cost-effective is 76% and the probability that CMA alone is cost-effective is 17%. As the WTP increases, the probability that the stepwise is cost-effective becomes closer to 100%.<br>*Phenotype: Abdominal/Gastro*<br>Figure S14 presents the CEAC for the subgroup associated with abdominal and gastro anomalies. At a WTP of £20,000 (€22,923/$25,654) the probability that the stepwise is cost-effective is approximately 20% and the probability that CMA alone is cost-effective is 80%. At a WTP of £30,000 (€34,385/$38,481) the probability that the stepwise cost-effective is 32% and the probability that CMA alone is cost-effective is 67%. If the WTP is greater than £50,000 (€57,308/ $64,135), the probability that the stepwise is cost-effective will exceed the probability that CMA alone is cost-effective.<br>*Phenotype: Nuchal Translucency*<br>Figure S15 presents the CEAC for the subgroup associated with nuchal translucency anomalies. At a WTP of £20,000 (€22,923/$25,654) the probability that the stepwise is cost-effective is 20% and the probability that CMA alone is cost-effective is 61%. At a WTP of £30,000 (€34,385/$38,481) the probability that the stepwise cost-effective is 40% and the probability that CMA alone is cost-effective is 35%.<br>*Phenotype: Brain*<br>Figure S16 presents the CEAC for the subgroup associated with brain anomalies. At a WTP of £20,000 (€22,923/$25,654) and £30,000 (€34,385/$38,481), the probability that the stepwise is cost-effective is 0% and the probability that CMA alone is cost-effective is 100%. There is no threshold whereby the probability of cost-effectiveness changes. This is because the only difference between each strategy was the cost.

Figure S1. Model Decision Tree

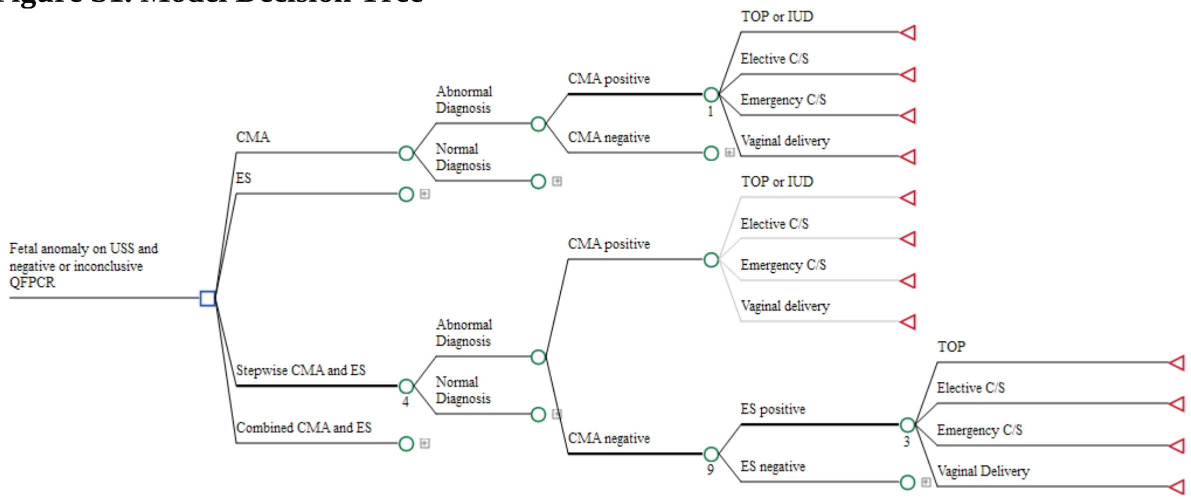

The decision tree was built for the purpose of the study. The branch structure for CMA (Chromosomal Microarray), 'Normal Diagnosis', follows the same structure as the 'Abnormal Diagnosis'. That is, after the diagnosis has been defined by QF-PCR and USS, the inidivudual moves to their CMA tests, which can be either positive or negative. The patient finally reaches their pregnancy outcome, which can be: 1. Termination of Pregnancy (TOP)/ Intrauterine Demise (IUD) 2.Elective Caesarean (C/S) 3. Emergency C/S 4. Vaginal Delivery. The same branch structure is been applied to Exome Sequencing (ES) alone.

The branch structure for the Stepwise, 'Normal Diagnosis', follows the same structure as the 'Abnormal Diagnosis'. That is, after the diagnosis has been defined by QF-PCR and USS, the inidivudual moves to their CMA tests, which can be either positive or negative. If the individual has a positive test result, no further tests are undertaken and the patient proceeds to their pregnancy outcome (TOP/IUD, Elective C/S, Emergency C/S; Vaginal Delivery). If a the CMA test result reads negative, the individual is required to undertake an ES test. The results of the ES test can be either positive of negative. In either case, once the result has been confirmed, the patient moves to their pregnancy outcome.

Figure S2. ***A1:*** **Incremental Cost Effectiveness Plane: ES and Stepwise**

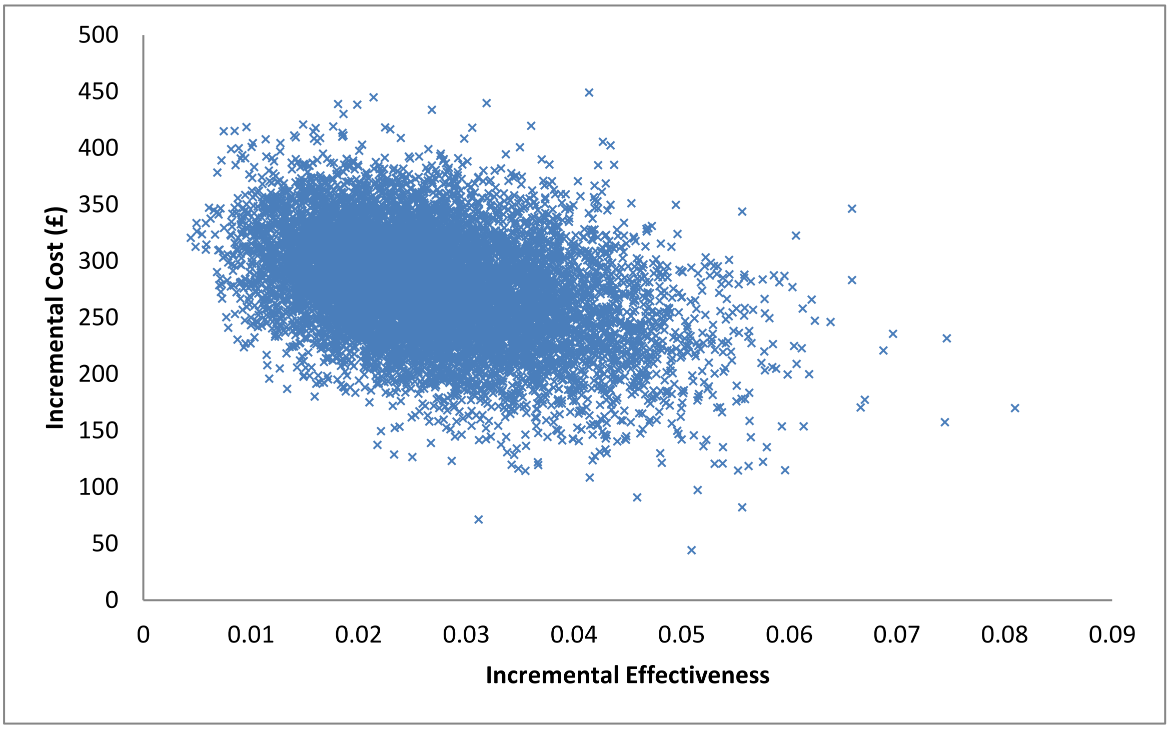

Figure S3. ***A2:*** **Cost Effectiveness Analysis, ES: £2,100**

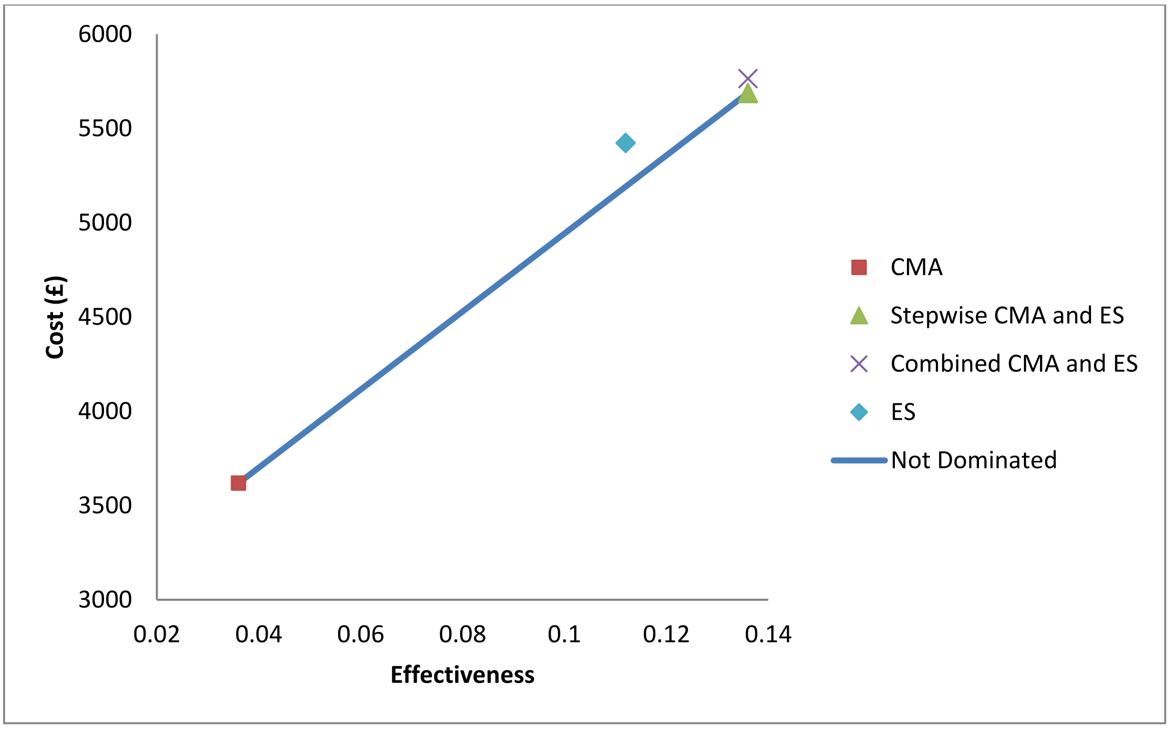

Figure S4. ***A2:*** **Cost Effectiveness Analysis, ES: £1,050**

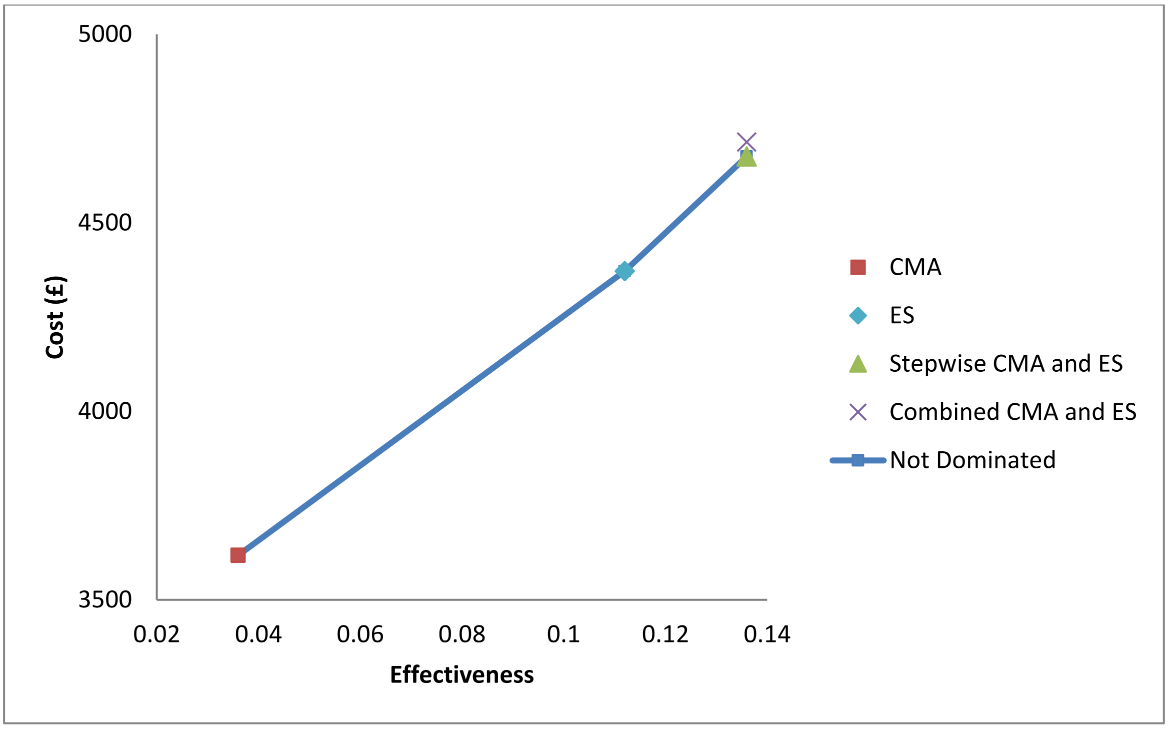

Figure S5. ***A2:*** **Incremental Cost Effectiveness Plane: CMA and ES**

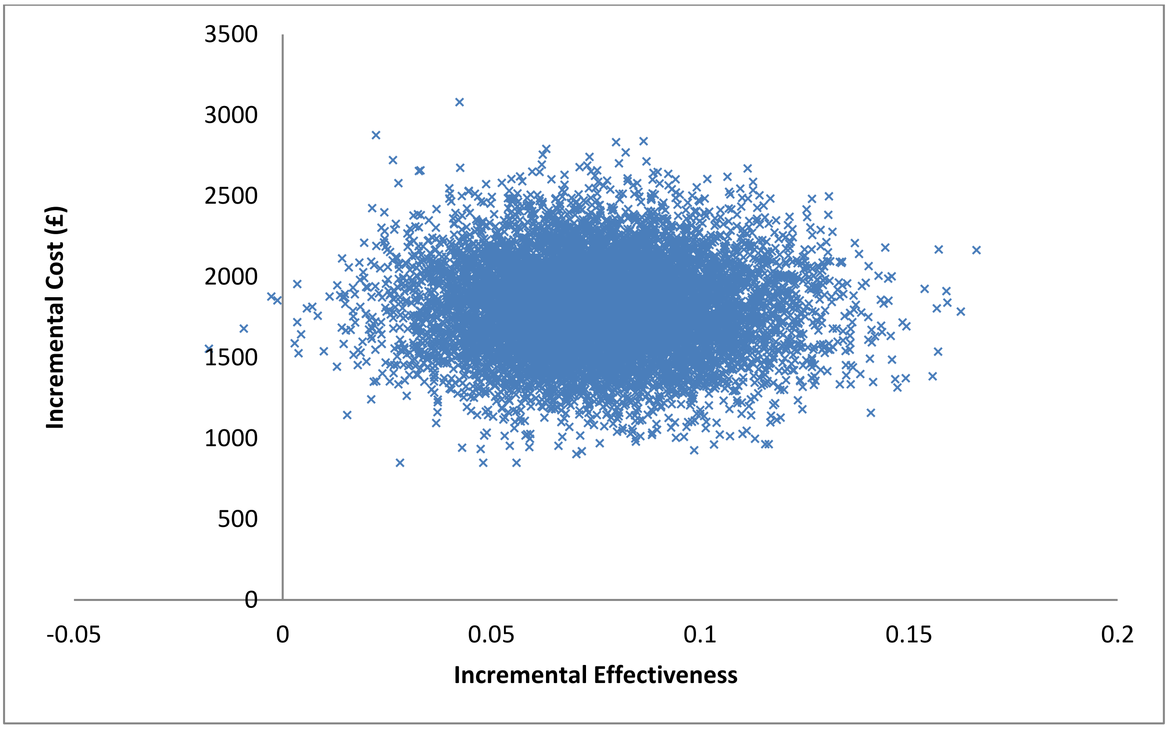

Figure S6. ***A2:* Incremental Cost Effectiveness Plane: CMA and Stepwise**

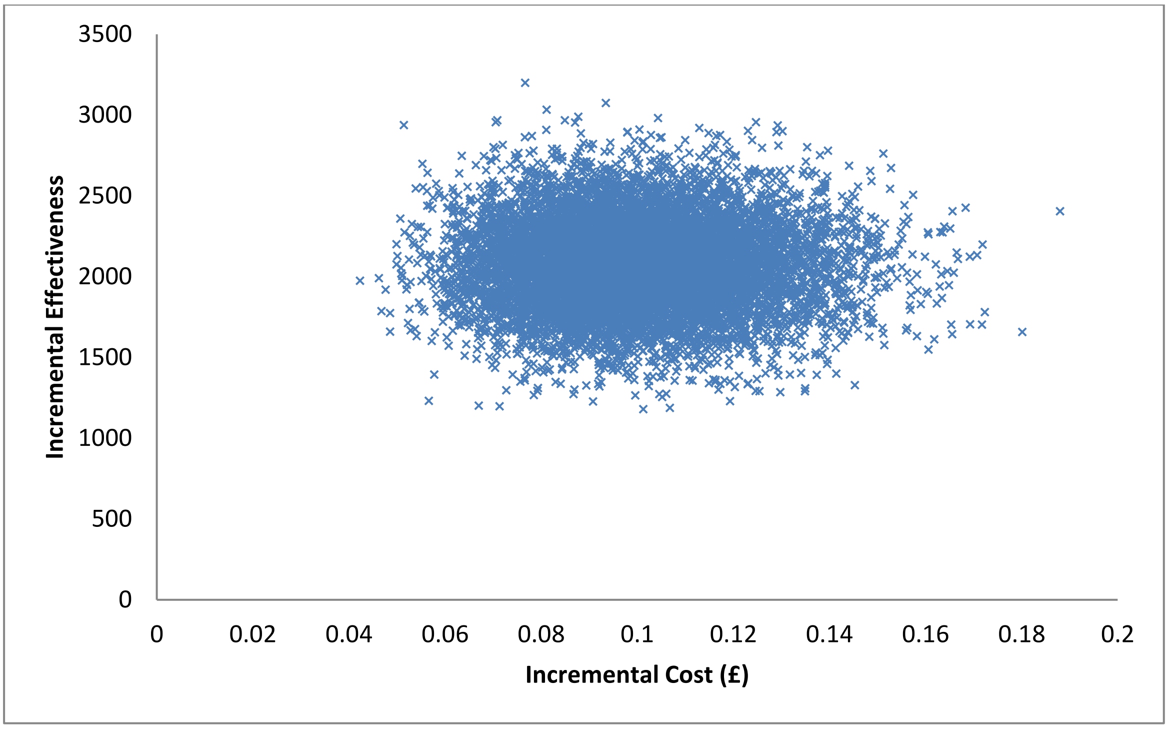

Figure S7. ***A2:* Incremental Cost Effectiveness Plane: ES and Stepwise**

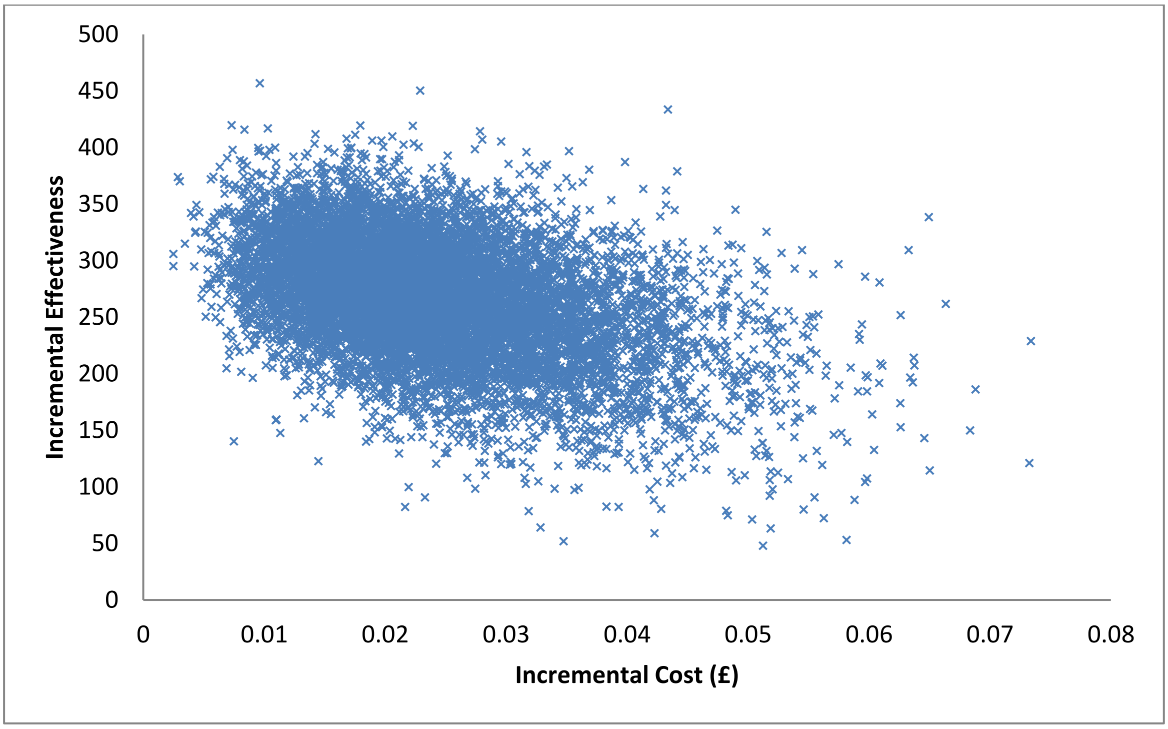

Figure S8. ***A2:*** **Cost Effectiveness Acceptability Curve (ES: £2100)**

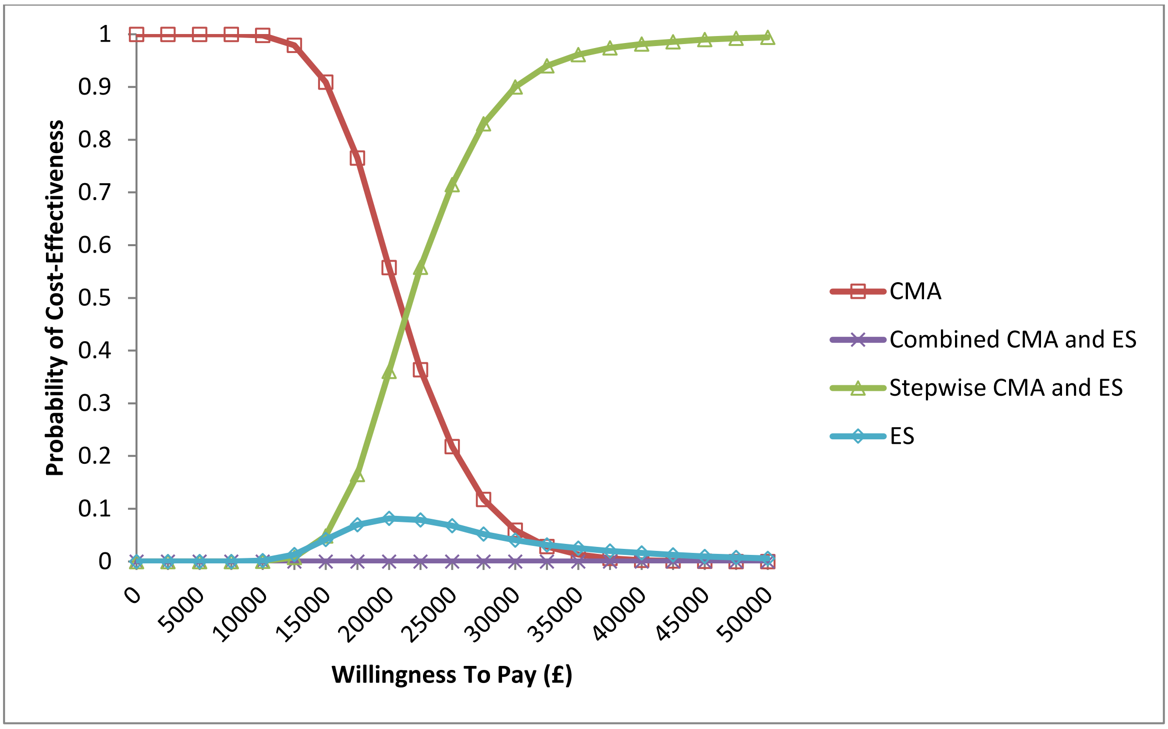

Figure S9. ***A2:*** **Cost Effectiveness Acceptability Curve (ES: £1050)**

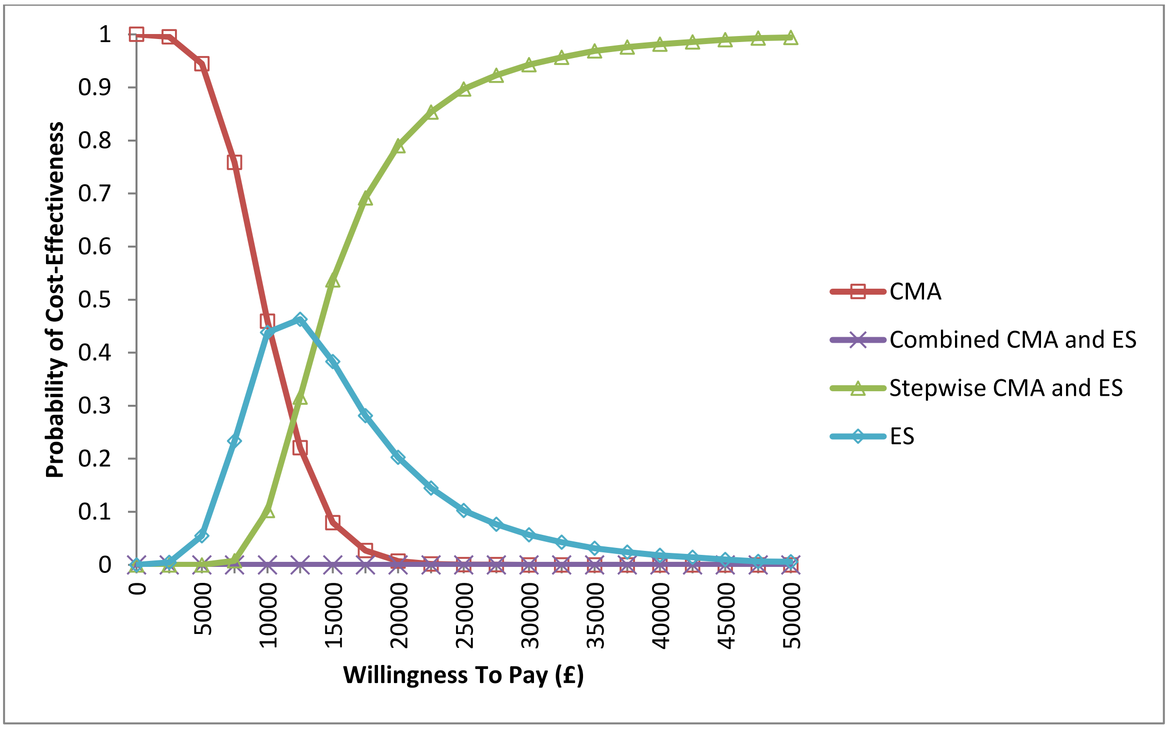

Figure S10. Cost Effectiveness Acceptability Curve for Multiple Anomalies (ES: £2,100)

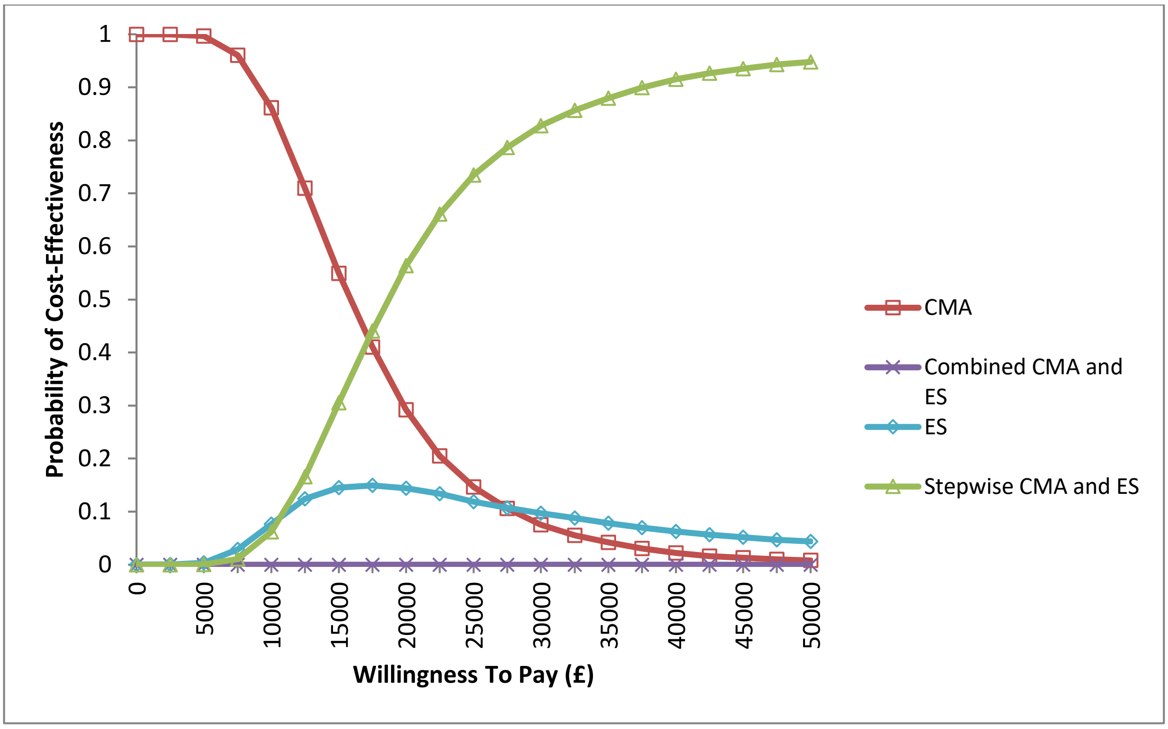

Figure S11. Cost Effectiveness Acceptability Curve for Single Anomalies (ES: £2,100)

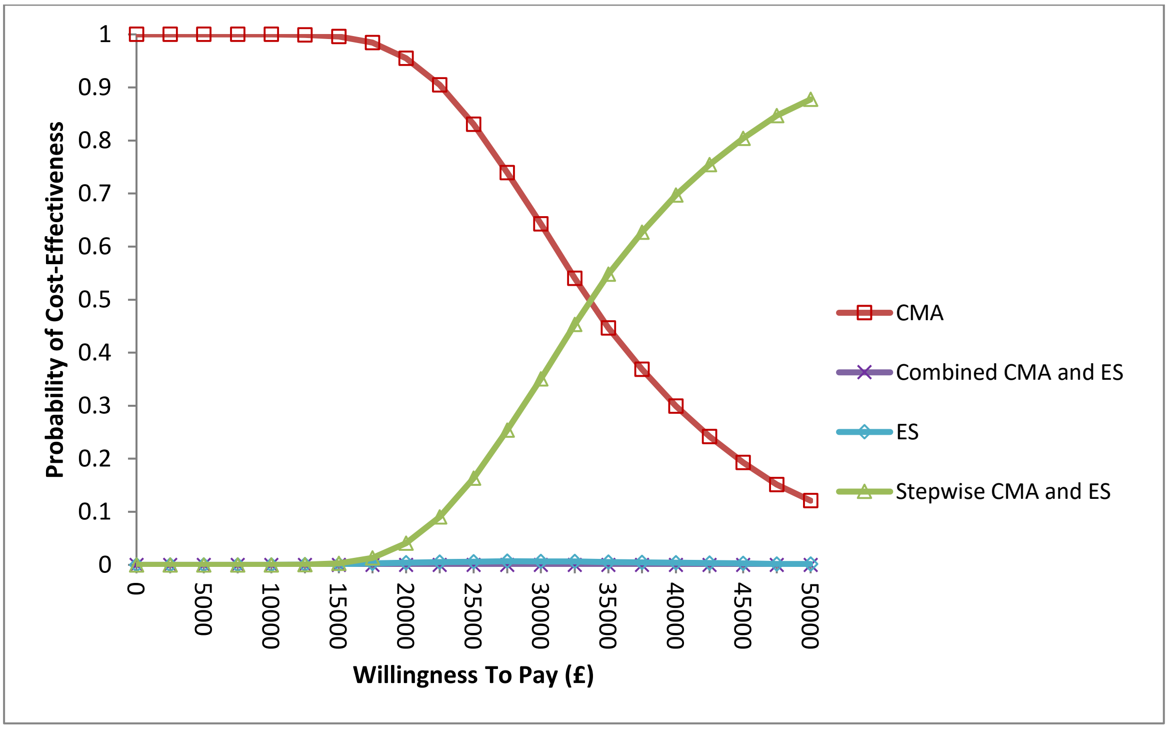

Figure S12. Cost Effectiveness Acceptability Curve for Skeletal, Limb and Spinal (ES: £2100)

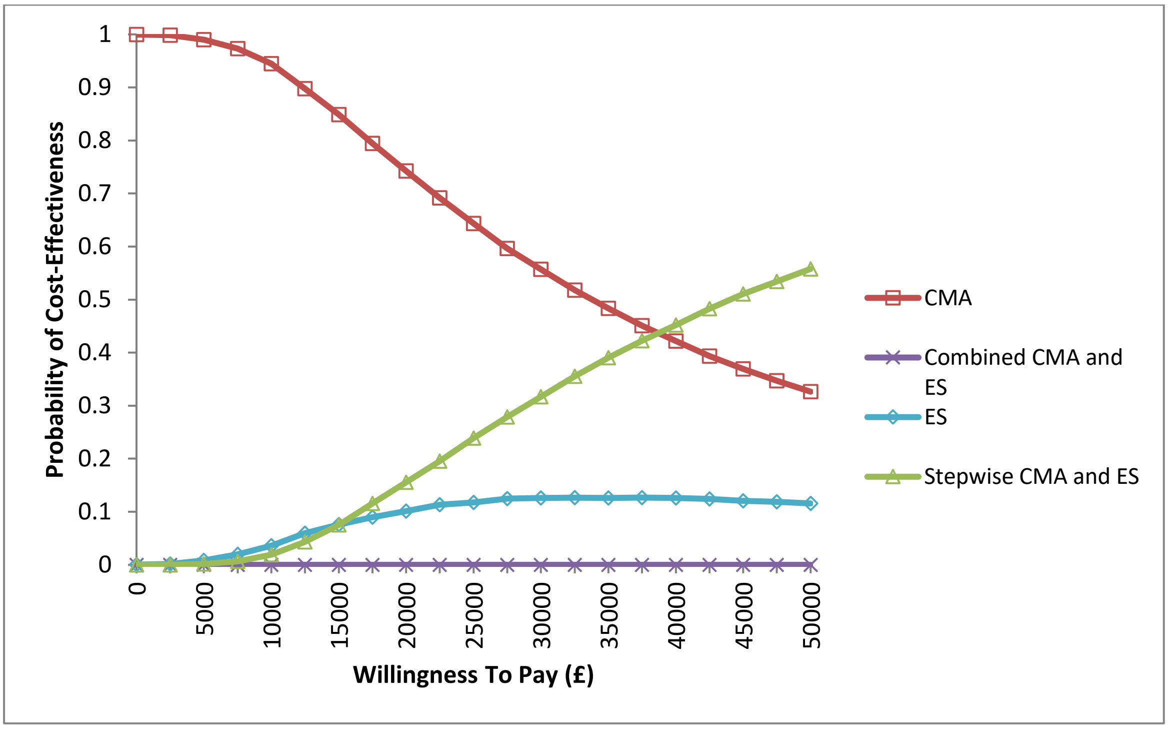

Figure S13. Cost Effectiveness Acceptability Curve for Cardiac (ES: £2100)

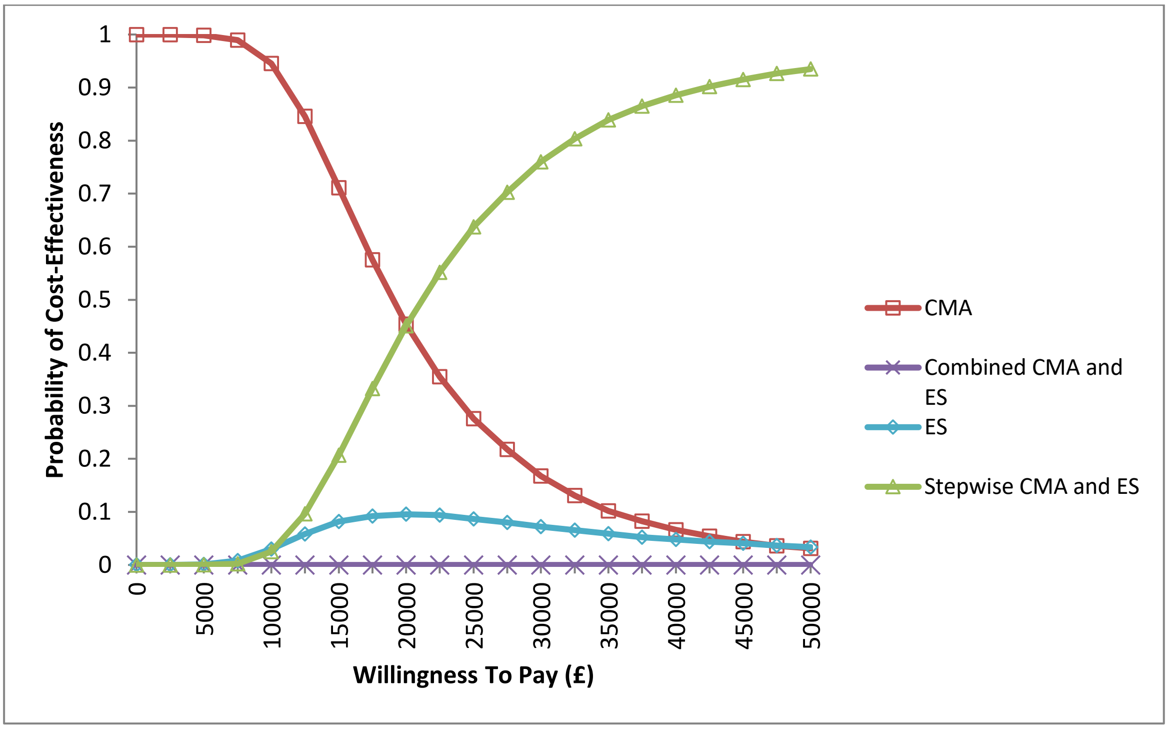

Figure S14. Cost Effectiveness Acceptability Curve for Abdominal and Gastro (ES: £2100)

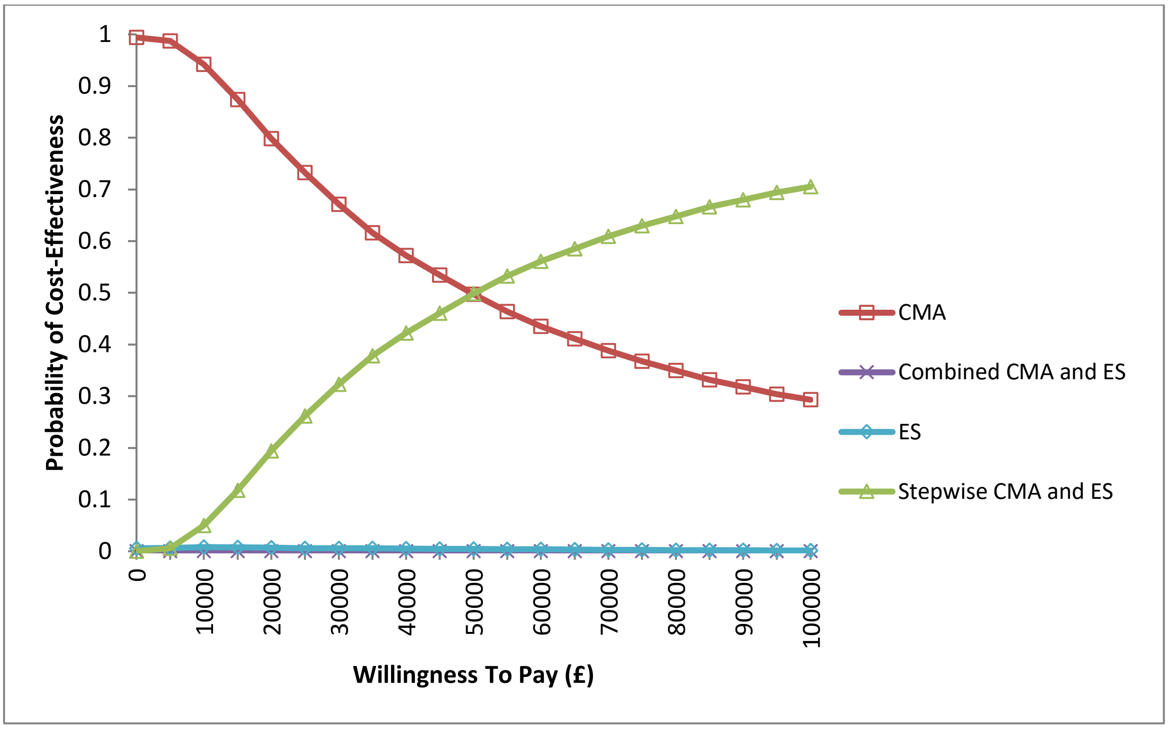

Figure S15. Cost Effectiveness Acceptability Curve for Nuchal Translucency (ES: £2100)

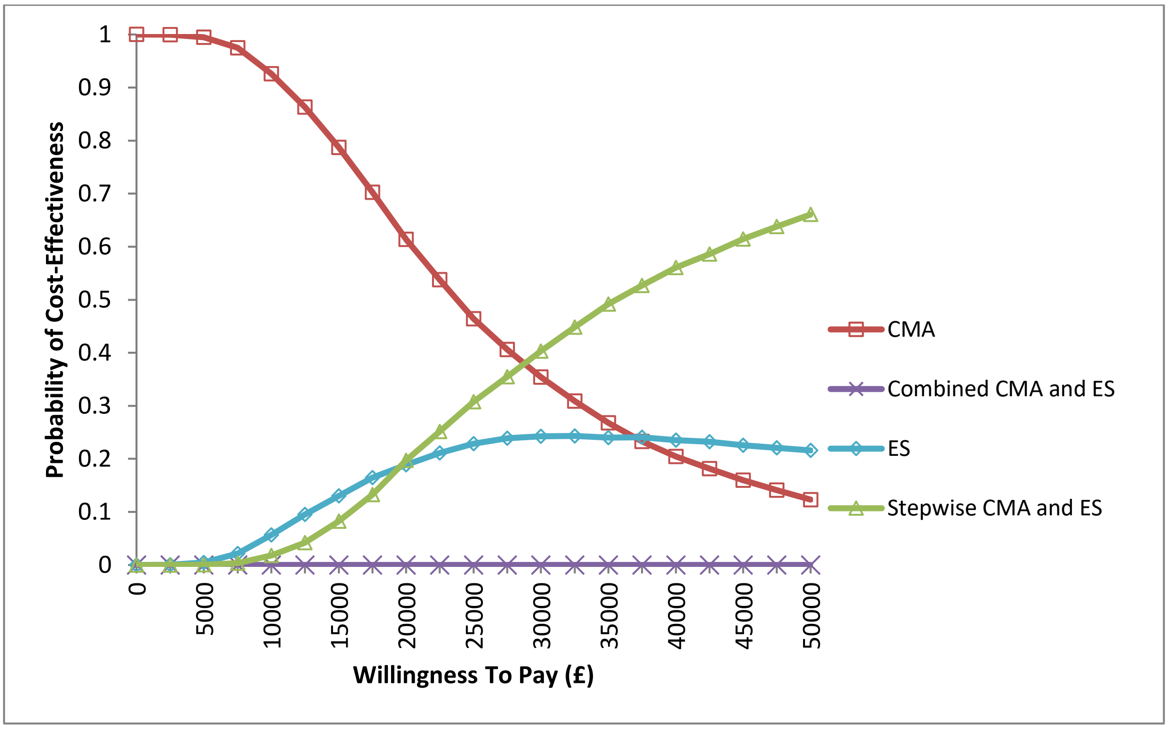

Figure S16. Cost Effectiveness Acceptability Curve for Brain (ES: £2100)

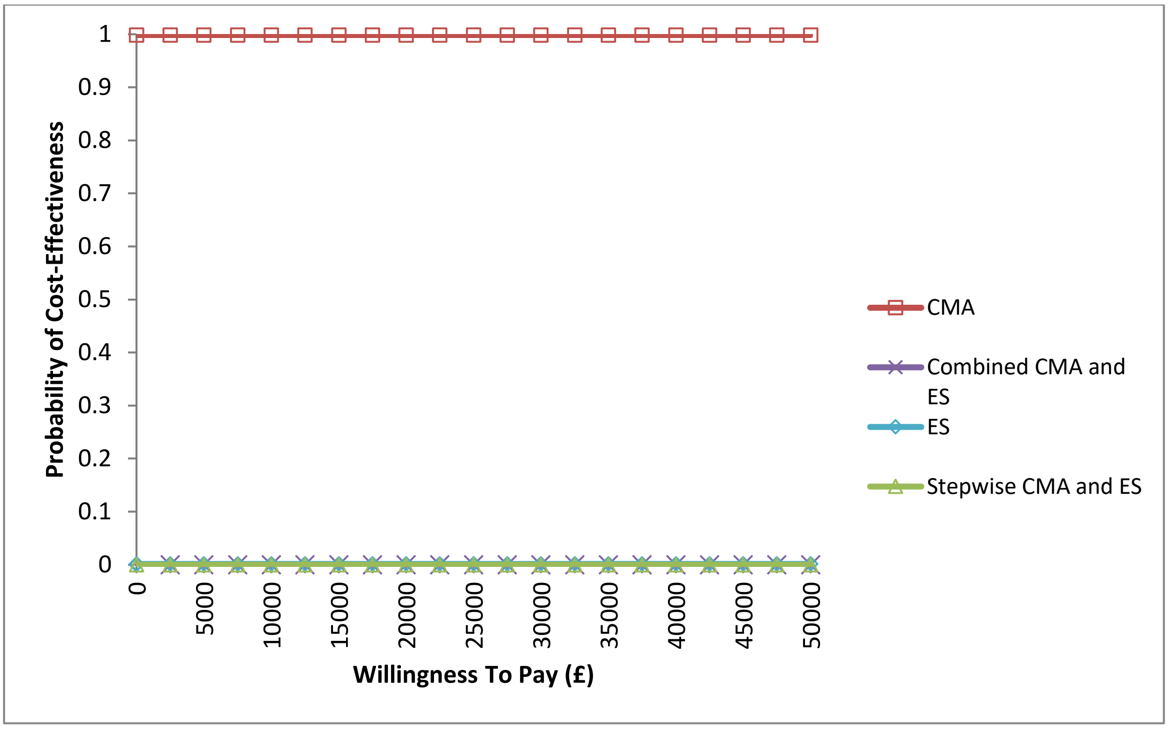

Supplement: Supplementary file 1 — Supplementary data [file fdt-0047-0554-s01.pdf]
